# Oxymatrine for inflammatory bowel disease in preclinical studies: a systematic review and meta-analysis

**DOI:** 10.3389/fmed.2025.1542953

**Published:** 2025-04-30

**Authors:** Xuan Zhao, Xiaolu Ye, Yuting Gu, Yijie Lou, Zhanyi Zhou, Yunxi Ji, Daogun Xu

**Affiliations:** ^1^The First Clinical College of Zhejiang Chinese Medical University, Hangzhou, Zhejiang, China; ^2^The First Affiliated Hospital of Zhejiang Chinese Medical University, Hangzhou, Zhejiang, China; ^3^Wenling Hospital of Traditional Chinese Medicine Affiliated to Zhejiang Chinese Medical University, Taizhou, Zhejiang, China

**Keywords:** oxymatrine, inflammatory bowel disease, meta-analysis, inflammation, oxidative stress

## Abstract

**Background:**

Inflammatory Bowel Disease (IBD) is a chronic, idiopathic inflammatory disorder of the intestines. Oxymatrine (OMT) is a naturally active substance found in the desiccated roots of *Sophora flavescens*. It possesses anti-tumor, antiviral, and anti-inflammatory properties. In recent years, its therapeutic role in IBD has gradually been discovered. This review aims to explore the impact of OMT on inflammatory bowel disease by animal models.

**Methods:**

Conduct a systematic search in the PubMed, Embase, Web of Science, Cochrane, and Medline databases. Using SYRCLE’s risk of bias tool to assess the bias risk and quality of the included studies. For some data presented as figures, Web Plot Digitizer 4.2 software was used to extract it. STATA 16.0 was selected for the final meta-analysis.

**Results:**

After rigorous literature screening, 12 studies were included. The data analysis results indicated that the disease activity index (DAI), histopathological score (HS), interleukin-6 (IL-6), interleukin-1β (IL-1β), tumor necrosis factor-*α* (TNF-α), nuclear factor-κB (NF-κB), and myeloperoxidase (MPO) activity in the IBD animal models significantly decreased following intervention with oxymatrine. Furthermore, OMT also extended the colon length in the animal models and improved the expression level of zonula occludens-1(ZO-1) and occludin. These results suggested that OMT may improve the condition of IBD through anti-inflammatory, antioxidative stress and protecting the intestinal barrier.

**Conclusion:**

Meta-analysis suggests oxymatrine positively affects IBD animal models. This provides new insights for the clinical treatment of inflammatory bowel disease.

**Systematic review registration:**

https://www.crd.york.ac.uk/PROSPERO/view/CRD42024570580, identifier [CRD42024570580].

## 1 Introduction

Inflammatory bowel disease (IBD), encompassing ulcerative colitis (UC) and Crohn’s disease (CD), is a chronic inflammatory condition of the gastrointestinal tract with an unclear etiology (1). IBD has complex pathogeneses, which involve factors like environment, genetics, immune response, intestinal barrier function, and gut microbiota. Although immunosuppressants, aminosalicylic acid and corticosteroids are routinely used in the treatment of IBD, they often fail to maintain long-term symptom relief and may lead to some side effects and complications (2, 3). More than 10% of patients eventually require surgical intervention due to the high failure rate of existing drugs (4). As a global disease, IBD has seriously affected the quality of patients’ life and brought a huge social burden (5). Therefore, there is an urgent need to develop more effective and safer therapeutic options for IBD.

Natural bioactive compounds have garnered attention for their potential therapeutic benefits in various diseases due to their efficacy and safety profiles (6). Oxymatrine (OMT) is a natural quinoline alkaloid extracted from *Sophora flavescens* in traditional Chinese medicine (7). Existing studies have confirmed that OMT possesses pharmacological effects such as anti-inflammatory, antibacterial, immunomodulatory, and antioxidant properties (8). OMT has demonstrated therapeutic potential in cardiovascular diseases, diabetes, hepatitis B, and cancers (9–14). In the context of IBD, several studies have explored the effects of OMT on intestinal inflammation. Experimental evidence indicates that OMT alleviates dextran sulfate sodium (DSS)-induced colitis by modulating the PI3K/AKT signaling pathway, leading to reduced inflammation and tissue damage (15). OMT has also been shown to repair the intestinal immune system by increasing CD4+ T cells in colonic tissues and reducing the secretion of pro-inflammatory cytokines (16). Additionally, recent research suggests that OMT attenuates UC by inhibiting ferroptosis and inflammation, targeting the expression of IL-1β, IL-6, NOS2, HIF1A, IDO1, TIMP1, and DUOX2 (17). These findings highlight the multifaceted mechanisms through which OMT may exert protective effects against IBD.

Given the increasing number of preclinical studies demonstrating the potential benefits of OMT in IBD models, it is essential to synthesize these findings to gain a clearer understanding of its therapeutic value. To address this need, we conducted a meta-analysis of animal studies on the use of OMT in IBD, providing a consolidated evaluation of its efficacy and potential mechanisms. By analyzing and comparing differences across various drug dosages, administration methods, and administration time, we aim to offer practical guidance for optimizing the clinical application of OMT. Additionally, exploring the possible mechanisms of OMT action will provide direction for future research efforts. By filling the current gap in the literature, we hope to contribute valuable information that can guide clinical practice and inform subsequent studies, ultimately improving therapeutic outcomes for patients with IBD.

## 2 Methods

This review was designed and executed in alignment with the Preferred Reporting Items (PRISMA) guidelines and registered on PROSPERO (CRD42024570580) in August 2024.

### 2.1 Literature search strategy

We conducted a search across five electronic databases: PubMed, Embase, Web of Science, Cochrane, and Medline. The search covered literature from the database’s inception to July 23, 2024, with no language restrictions. A corresponding search strategy was developed based on the search rules of different databases. Relatively appropriate MeSH subject headings and free words were selected, such as: “Inflammatory Bowel Diseases,” “IBD,” “Colitis, Ulcerative,” “Ulcerative Colitis,” “Crohn Disease,” “oxymatrine,” “kurorinone,” etc. The Supplementary material S1 presents a detailed search strategy.

### 2.2 Inclusion and exclusion criteria

Studies were included according to the following criteria: (1) All animal models of inflammatory bowel diseases, regardless of species, sex, or age; (2) Investigate the efficacy of oxymatrine in animal models of IBD; (3) The treatment group only used oxymatrine (regardless of dosage form, dose, route of administration, and administration time), while the control group was not treated or was administrated with placebo; (4) Complete and available outcomes; (5) All animal controlled intervention study; (6)English literature.

Studies were excluded according to the following criteria: (1) Experiments did not use IBD animal models; (2) *In vitro* research; (3) The intervention was OMT combined with other medications; (4) Without control group; (5) Repeated published research; (6) Reviews, conference abstracts, human clinical studies, case reports and commentary type studies; (7) Unable to extract relevant outcomes.

### 2.3 Data collection

The studies’ data and information were separately collected by two authors, with any discrepancies settled by consulting a third reviewer (Y.T.G). The information extracted includes (1) Title and publication year; (2) First author and country; (3) Animal strain, age, weight, and sex; (4) Modeling methods and modeling time of IBD animal models; (5) Experimental grouping; (6) Oxymatrine administration (dose, administration method, intervention time); (7) Follow-up duration; (8) Outcomes: Disease activity index (DAI), histopathological score (HS), colon length, interleukin-6 (IL-6), interleukin-1β (IL-1β), interleukin-10 (IL-10), tumor necrosis factor-*α* (TNF-α), nuclear factor-κB (NF-κB), myeloperoxidase (MPO) activity, zonula occludens-1 (ZO-1), occludin. The outcomes extracted are all continuous variables. We prioritized extracting numerical data from tables and text. If the data is only provided as a figure, we use the Web Plot Digitizer 4.2 software to extract the data.

### 2.4 Bias risk assessment of the study

The bias risk of each study was evaluated by two reviewers (X.Z; X.L.Y) using SYRCLE’s risk of bias tool (18). If there were discrepancies, a third reviewer (Y.T.G) was consulted to reach a consensus. The review was divided into ten items: sequence generation, baseline characteristics, allocation concealment, random housing, blinding of the study team, random outcome assessment, blinding of outcome assessors, incomplete outcome data, selective outcome reporting, and other biases. Rate each item as “Yes,” “No,” or “Unclear” to represent a low, high, or uncertain risk of bias. Specific risk assessment methods for each project can be found in the Supplementary material S2.

### 2.5 Statistical analysis

Using STATA 16.0 software to conducted the gathered data. We extracted the means and standard deviations (SD) from studies for analysis, with a 95% confidence interval (CI). Standard mean difference (SMD) was chosen for data with non-uniform units to represent effect sizes. Weighted mean difference (WMD) was used to represent data with uniform units. The I^2^ test was used to assess the magnitude of heterogeneity. I^2^ ≤ 50% indicates low heterogeneity, the fixed-effect model is used; I^2^ > 50% indicates high heterogeneity, and the random-effects model is used for data merging. Subgroup analysis was performed using six variables: animal species, model, dosage of OMT, time of administration, administration method, and follow-up duration. If a study comprises multiple experimental groups and a single control group, the total number of animals in the control group can be corrected by dividing the sample size of the control group by the number of experimental groups (19). Funnel plots were employed to evaluate publication bias for metrics. If publication bias was substantial, a trim-and-fill test was performed to assess whether this bias affected the outcomes. Sensitivity analysis was performed to evaluate the stability of the results for indicators with high heterogeneity.

## 3 Result

### 3.1 Search results

**Figure 1:**
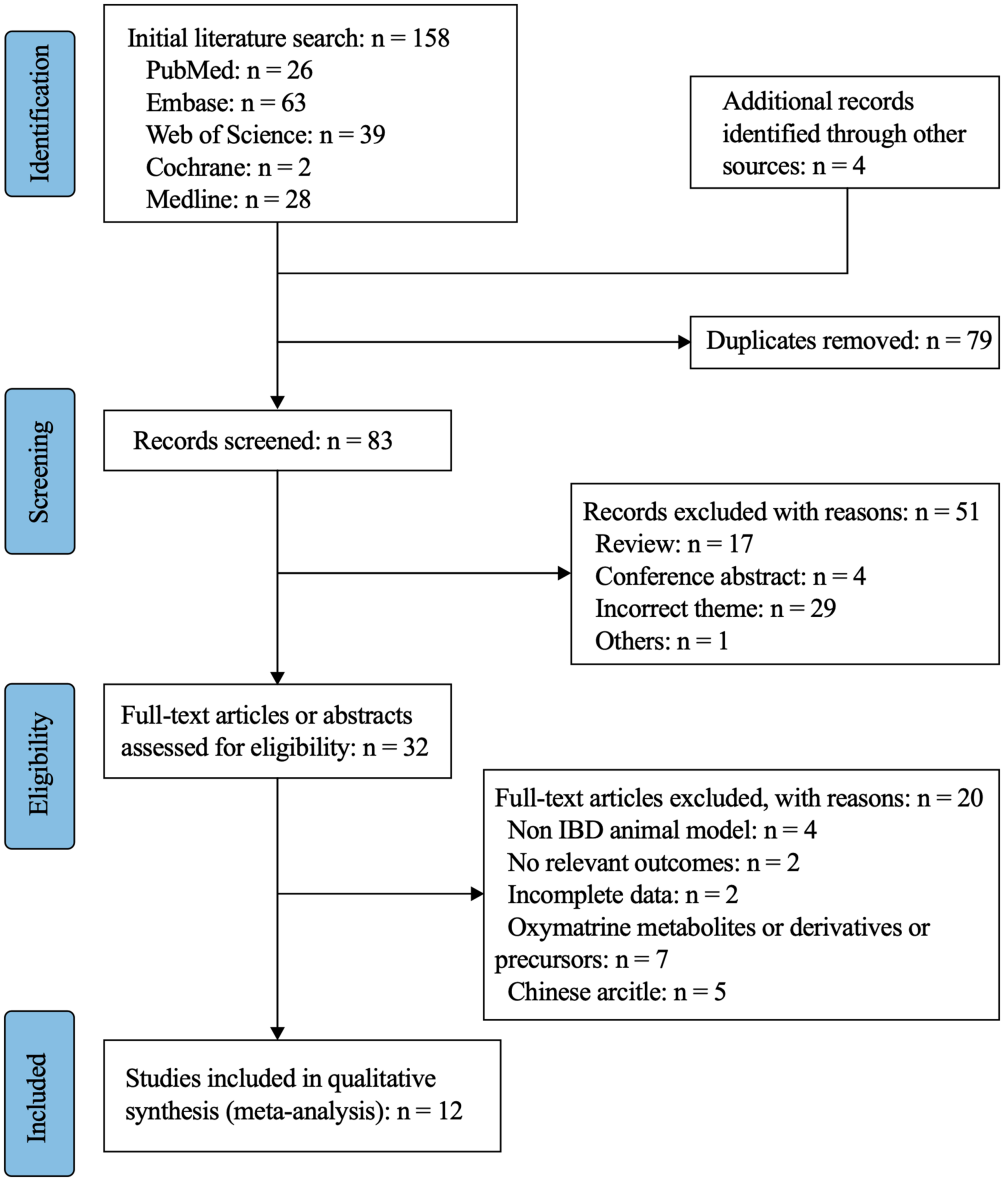
Flow diagram of the study selection.

162 documents were searched (Figure 1), of which 158 were obtained from databases, and the remaining 4 were added manually. After excluding 79 duplicates, 51 papers were further excluded on the basis of their titles and abstracts. Of these, 17 were reviews, 4 were conference abstracts, 1 was a bibliometric analysis, and 29 did not match the study content. Thirty-two articles were selected for full-text review, and 20 studies were excluded: 4 were non-IBD animal models (20–23), 2 lacked relevant outcome measures (24, 25), 2 lacked complete data (26, 27), 5 were written in Chinese (28–32), and 7 used oxaliplatin metabolites, derivatives, or precursors as the intervention (33–39). A total of 12 studies were included in the meta-analysis (7, 15–17, 40–47).

### 3.2 Features of the included studies

The review encompassed 12 studies from 2005 to 2024, containing 19 experiments. Of the included studies, 5 used rats, and 7 used mice. Most studies employed male animals as the model subjects, with only one study utilizing female mice. This may be because the immune system of male animals is more susceptible to certain stimuli, which enhances the experiment’s reproducibility. Regarding the modeling method, 8 studies used dextran sodium sulfate (DSS) to induce IBD, while 4 studies chose 2,4,6-trinitrobenzene sulfonic acid (TNBS). The modeling time using DSS was basically 6–8 days, with one study lasting 9 days and two studies lasting 14 days. TNBS was mainly administered rectally for modeling, and the modeling time is generally 24 h. Only one study was modeled for 7 days, but the administration method was not clearly stated. 4 studies compared interventions with different doses of OMT. The lowest dose of OMT used in the studies was 10 mg/kg, and the highest was 180 mg/kg. In addition, 2 studies chose to administer drugs by gavage, 4 studies utilized intramuscular injection, 5 studies employed intraperitoneal administration, and 1 study did not specify the method of administration. All studies took place in China, except one from Japan. This may be related to the origin of pharmaceutical raw materials. Details of the included studies are shown in Table 1.

**Table 1:** Characteristics of the 12 studies included in the meta-analysis.

Study	Animal	model	Groups	Dosage of OMT	Administration method of OMT	Time of administration	Follow-up duration	Outcomes
Zheng et al. (47), China	Male SD rats, 4-5wk	2% DSS for 8 days	DSS + NS (*n* = 10)	63 mg/kg/day	Intramuscular injection	Before IBD induction	11 days	DAI, HS, TNF-*α*, IL-6, NF-κB
			DSS + OMT (*n* = 10)					
Fan et al. (40), China	Male SD rats, 180-225 g	TNBS was injected into the colon (24 h)	TNBS + NS (*n* = 10)	63 mg/kg/day	Intramuscular injection	After IBD induction	15 days	HS, NF-κB, IL-10
			TNBS + OMT (*n* = 10)					
Fan et al. (41), China	Male SD rats, 250 g ± 20 g	TNBS was injected into the colon (24 h)	TNBS + water (*n* = 10)	63 mg/kg/day	Intramuscular injection	After IBD induction	15 days	HS, NF-κB
			TNBS + OMT (*n* = 10)					
Zhou et al. (16), China	Male SD rats, 200-250 g	TNBS was injected into the colon (24 h)	TNBS + water (*n* = 10)	63 mg/kg/day	Intramuscular injection	After IBD induction	15 days	HS
			TNBS + OMT (*n* = 10)					
Chen et al. (15), China	Male BALB/c mice, 18-22 g	3% DSS for 7 days	DSS + PBS	/	Intraperitoneal injection	From the first day of the model induction	7 days	Colon length, HS
			DSS + Low-dose OMT	25 mg/kg/day				
			DSS + Moderate-dose OMT	50 mg/kg/day				
			DSS + High-dose OMT	100 mg/kg/day				
Wang et al. (44), China	Male BALB/c mice, 18-22 g	3% DSS for 7 days (acute)	DSS + PBS (*n* = 8)	/	Intraperitoneal injection	From the first day of the model induction	7 days	Colon length, DAI, HS, IL-10, MPO
			DSS + Low-dose OMT (*n* = 8)	25 mg/kg/day				
			DSS + Moderate-dose OMT (*n* = 8)	50 mg/kg/day,				
			DSS + High-dose OMT (*n* = 8)	100 mg/kg/day				
Tang et al. (43), China	Male mice,18 ± 2 g	3% DSS for 7 days (acute)	DSS + saline (*n* = 6)	20 mg/kg/day	Intraperitoneal injection	After IBD induction	7 days	Colon length, DAI, TNF-α, IL-6, IL-1β, MPO
			DSS + OMT (*n* = 6)					
Zhang et al. (45), Japan	Male BALB/c mice, 8wk	3% DSS for 7 days	DSS + saline	100 mg/kg/day	Intraperitoneal injection	From the first day of the model induction	7 days	Colon length, DAI
			DSS + OMT					
Li et al. (42), China	Male SD rats, 190 ± 20 g	TNBS for 7 days	TNBS + PBS (*n* = 10)	/	Intraperitoneal injection	From the first day of the model induction	7 days	Colon length, HS, TNF-α, IL-6, IL-1β, NF-κB, IL-10
			TNBS + Low-dose OMT (*n* = 10)	10 mg/kg/day				
			TNBS + Moderate-dose OMT (*n* = 10)	30 mg/kg/day				
			TNBS + High-dose OMT (*n* = 10)	60 mg/kg/day				
Liu et al. (7), China	Male BALB/c mice, 22 ± 2 g	3% DSS for 7 days, water for 7 days, 2% DSS for 7 days	DSS + sterile water (*n* = 10)	100 mg/kg/day	Intragastric gavage	After IBD induction	21 days	Colon length, DAI, HS, NF-κB
			DSS + OMT (*n* = 10)					
Gao et al. (17), China	Female ICR mice, 8wk	4% DSS for 14 days	DSS + sterile water (*n* = 8)	/	/	From the first day of the model induction	14 days	Colon length, TNF-α, IL-6, IL-1β
			DSS + Low-dose OMT (*n* = 8)	50 mg/kg/day				
			DSS + High-dose OMT (*n* = 8)	100 mg/kg/day				
Zhao et al. (46), China	Male BALB/c mice, 18-22 g	10% DSS twice daily for 9 days	DSS + distilled water (*n* = 5)	180 mg/kg/day	Intragastric gavage	After IBD induction	9 days	Colon length, DAI, HS, TNF-α, IL-6, IL-1β, IL-10, MPO
			DSS + OMT (*n* = 5)					

DSS, dextran sodium sulfate; TNBS, 2,4,6-trinitrobenzenesulfonic acid; NS, normal saline; OMT, oxymatrine; PBS, Phosphate Buffered Saline; IBD, Inflammatory Bowel Disease; DAI, disease activity index; HS, histopathological score; IL-6, interleukin-6; IL-10, interleukin-10; IL-1β, interleukin-1β; TNF-α, tumor necrosis factor-α; NF-κB, nuclear factor-κB; MPO, myeloperoxidase.

### 3.3 Bias risk assessment

The results are shown in Table 2. The 12 studies included in the review were subjected to a rigorous evaluation. The majority of studies showed low risk in 5–7 items, with an acceptable overall risk of bias. Only one research clearly stated that randomization was performed using a random number table. 8 studies reported baseline characteristics in each group. However, none of the included studies described the actual implementation of random allocation and blinding, so we could not determine whether allocation concealment and blinding were adequate. 5 studies described randomization of animal placement. For the measurement of outcome indicators, 3 studies referred to randomization of animal selection, and 6 implemented blinding. No selective reporting was found in any of the studies, and no instances of missing data were identified. We did not identify additional sources of bias after evaluation.

**Table 2:** SYRCLE’s risk of bias tool for each experimental animal study.

Study										
Item	Sequence generation	Baseline characteristics	Allocation concealment	Random housing	Blinding (study team)	Random outcome assessment	Blinding (outcome assessors)	Incomplete outcome data	Selective outcome reporting	Other bias
	Selection bias			Performance bias		Detection bias		Attrition bias	Reporting bias	Other
Zheng et al. (47), China	?	?	?	?	?	?	?	+	+	+
Fan et al. (40), China	?	+	?	?	?	?	+	+	+	+
Fan et al. (41), China	+	+	?	?	?	+	+	+	+	+
Tang et al. (29), China	?	+	?	?	?	?	+	+	+	+
Zhou et al. (16), China	?	+	?	+	?	+	+	+	+	+
Chen et al. (15), China	?	+	?	?	?	?	?	+	+	+
Wang et al. (44), China	?	+	?	+	?	?	?	+	+	+
Tang et al. (43), China	?	?	?	?	?	+	+	+	+	+
Zhang et al. (45), Japan	?	?	?	?	?	?	?	+	+	+
Li et al. (42), China	?	+	?	+	?	?	+	+	+	+
Liu et al. (7), China	?	+	?	+	?	?	+	+	+	+
Gao et al. (17), China	?	?	?	?	?	?	?	+	+	+
Zhao et al. (46), China	?	+	?	+	?	?	?	+	+	+

Yellow means “Unclear”, green means “Yes”.

### 3.4 Efficacy evaluation

#### 3.4.1 Final disease activity index score

**Figure 2:**
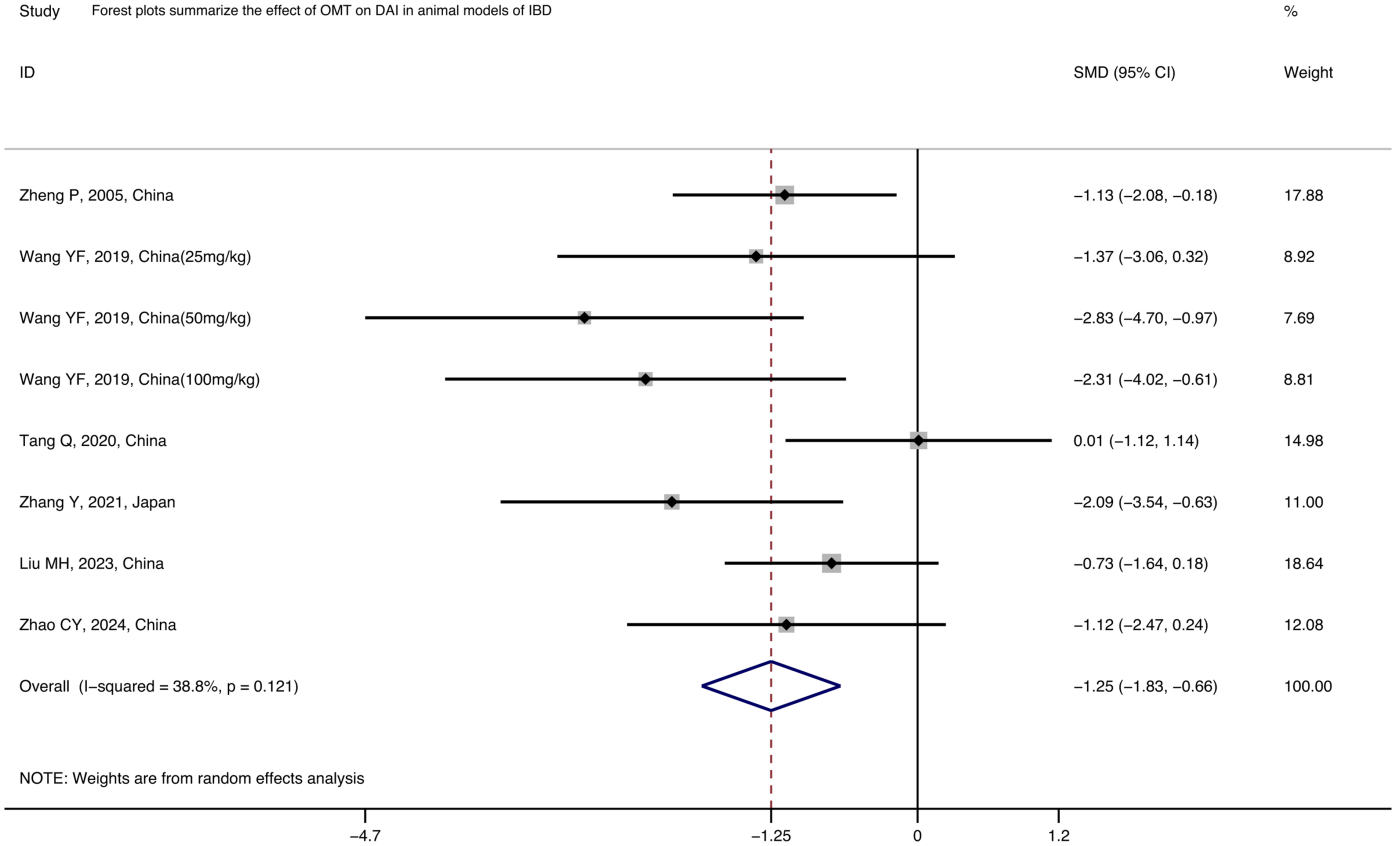
Forest plots summarize the effect of OMT on DAI in animal models of IBD.

Among the 12 studies included in the analysis, 6 reported final DAI score (7, 43–47) to assess the severity of IBD. It is comprehensively scored by evaluating the weight loss percentage in mice, stool consistency, and blood in stools. After data integration and analysis, the results indicated that OMT significantly reduced the DAI score compared to the model group [*n* = 61/45, SMD = −1.25 (−1.83, −0.66), *p* < 0.001, I2 = 38.8%; Figure 2].

Subgroup analysis of the DAI score was conducted based on five variables: animal species, dosage of OMT, time of administration, administration method, and follow-up duration (Supplementary Figure 1). Since all the studies used DSS for modeling, we did not perform a subgroup analysis based on the modeling method. Notably, the improvement in DAI score with a low dosage (< 30 mg/kg) of OMT in mice was not statistically significant (*p* = 0.429). Whereas compared with high-dose (> 60 mg/kg) OMT [5 studies, *n* = 39/34, SMD = −1.24 (−1.77, −0.70), *p* < 0.001; Table 3], the moderate-dose (30–60 mg/kg) OMT can significantly control DAI score in IBD mice [1 studies, *n* = 8/3, SMD = −2.83 (−4.70, −0.97), *p* = 0.003; Table 3]. However, due to the limited number of studies included for the moderate-dose OMT, it is challenging to judge that the moderate-dose OMT has better efficacy. In addition, administration from the first day of model induction [2 studies, *n* = 30/14, SMD = −2.12 (−2.95, −1.29), *p* < 0.001; Table 3] can lead to better control of the condition. This may suggest that administering oxymatrine treatment in the early stages of the disease could yield better efficacy.

**Table 3:** Subgroup analysis of the disease activity index.

Analysis	Trials (*n*)	Analyzed (E/C)	SMD (95% CI)	*p*-value	I^2^
Overall	8	61/45	−1.25 [−1.83, −0.66]	<0.001	38.8%
Animal species					
Rat	1	10/10	−1.13 [−2.08, −0.18]	0.020	–
Mice	7	51/35	−1.31 [−2.03, −0.59]	<0.001	47.5%
Dosage of OMT					
< 30 mg/kg	2	14/8	−0.53 [−1.86, 0.79]	0.429	43.7%
30-60 mg/kg	1	8/3	−2.83 [−4.70, −0.97]	0.003	–
> 60 mg/kg	5	39/34	−1.24 [−1.77, −0.70]	<0.001	2.9%
Time of administration					
Before IBD induction	1	10/10	−1.13 [−2.08, −0.18]	0.020	–
From the first day of the model induction	4	30/14	−2.12 [−2.95, −1.29]	<0.001	0.0%
After IBD induction	3	21/21	−0.59 [−1.21, 0.04]	0.068	0.0%
Administration method					
Intramuscular injection	1	10/10	−1.13 [−2.08, −0.18]	0.020	–
Intraperitoneal injection	2	12/12	−0.99 [−3.04, 1.07]	0.346	79.9%
Intragastric gavage	5	39/23	−1.43 [−2.18, −0.68]	<0.001	27.8%
Follow-up duration					
< 10 days	6	41/25	−1.49 [−2.37, −0.61]	0.001	51.4%
≥ 10 days	2	20/20	−0.92 [−1.58, −0.26]	0.006	0.0%

DAI, disease activity index; OMT, oxymatrine; SMD, standard mean difference; CI, confidence interval; E/C, experiment/control; IBD, inflammatory bowel disease.

#### 3.4.2 Histopathological score

**Figure 3:**
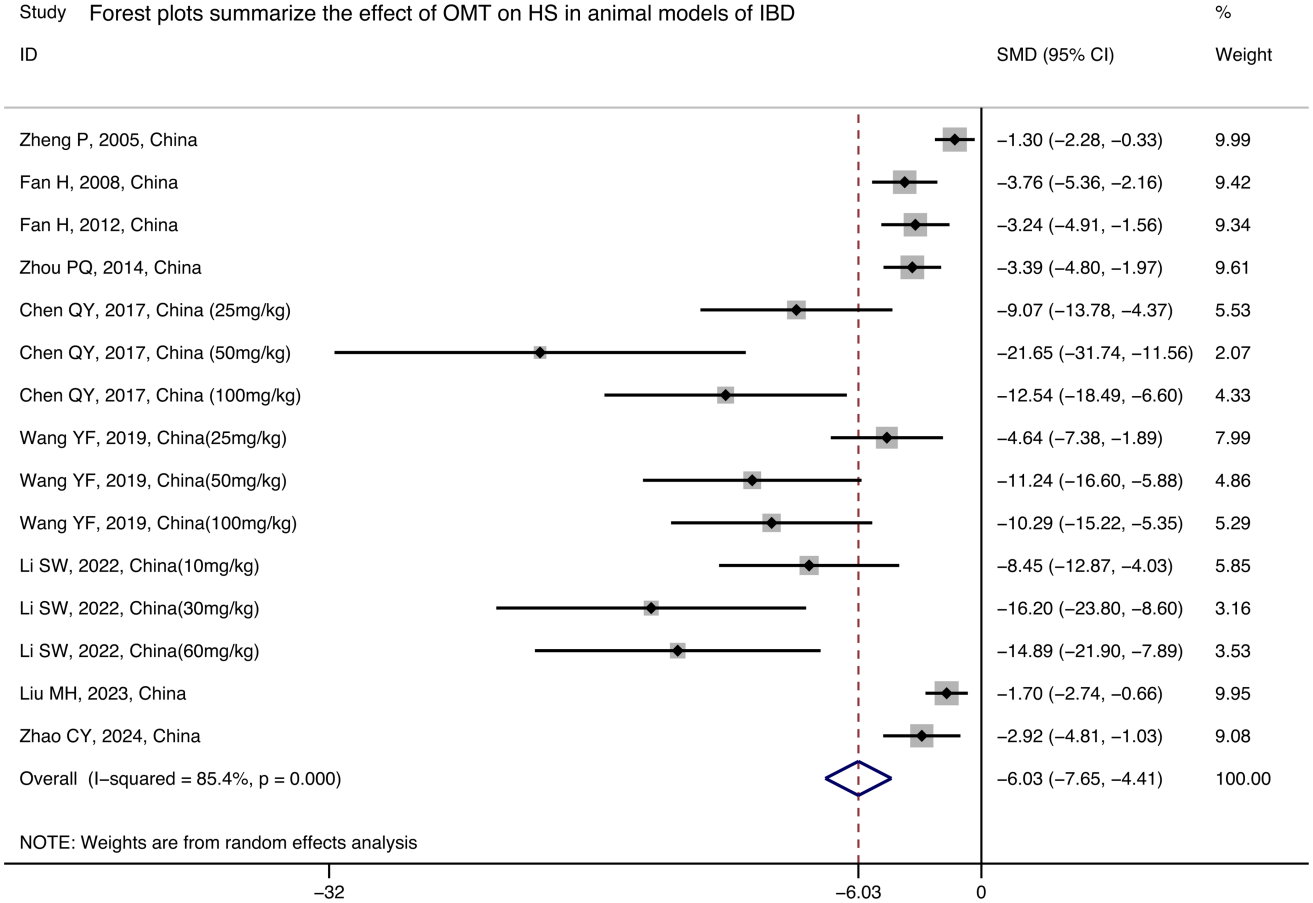
Forest plots summarize the effect of OMT on HS in animal models of IBD.

9 studies measured the histopathological score (7, 15, 16, 40–42, 44, 46, 47), which is an index that assesses the severity of inflammation in terms of histopathologic changes in the colon. The results showed that HS can be effectively controlled after treatment with OMT [*n* = 125/73, SMD = −6.03 (−7.65, −4.41), *p* < 0.001, I^2^ = 85.4%; Figure 3].

9 studies were included with HS as an outcome indicator, and subgroup analyses were performed based on different variables (Supplementary Figure 2). A comparison between subgroups showed no significant difference in the effect of varying modeling methods on HS. Nevertheless, a discernible distinction was observed between the different administration methods. In comparison to the other methods, with intraperitoneal injection [3 studies, *n* = 72/24, SMD = −10.99 (−14.05, −7.92), *p* < 0.001; Table 4] being able to minimize HS to a more significant reduction than the other methods. Interestingly, the results showed that OMT improved inflammation more significantly in mice [4 studies, *n* = 63/31, SMD = −7.68 (−10.75, −4.61), *p* < 0.001; Table 4] than in rats. In the same way as the results of the previous analyses, administration of the drug from the first day of the model induction (3 studies, *n* = 72/24, SMD = −10.99 (−14.05, −7.92), *p* < 0.001; Table 4) resulted in a better improvement in HS. The analysis of this subgroup revealed that the moderate-dose [3 studies, *n* = 32/12, SMD = −14.66 (−18.53, −10.80), *p* < 0.001; Table 4] OMT had the most significant improvement on HS. This may suggest that the optimal dose of OMT for mice/rats is between 30 mg/kg and 60 mg/kg.

**Table 4:** Subgroup analysis of the histopathological score.

Analysis	Trials (*n*)	Analyzes (E/C)	SMD (95% CI)	*p*-value	I^2^
Overall	15	125/73	−6.03 [−7.65, −4.41]	<0.001	85.4%
Animal species					
Rat	7	62/42	−4.99 [−7.08, −2.90]	<0.001	85.4%
Mice	8	63/31	−7.68 [−10.75, −4.61]	<0.001	87.0%
Models					
DSS	9	73/41	−6.19 [−8.47, −3.91]	<0.001	87.2%
TNBS	6	52/32	−5.95 [−8.31, −3.59]	<0.001	79.7%
Dosage of OMT					
< 30 mg/kg	3	24/6	−6.88 [−9.90, −3.87]	<0.001	45.1%
30-60 mg/kg	4	32/12	−14.66 [−18.53, −10.80]	<0.001	15.5%
> 60 mg/kg	8	69/55	−3.43 [−4.74, −2.12]	<0.001	79.1%
Time of administration					
Before IBD induction	1	10/10	−1.30 [−2.28, −0.33]	0.009	–
From the first day of the model induction	9	72/24	−10.99 [−14.05, −7.92]	<0.001	67.0%
After IBD induction	5	43/39	−2.87 [−3.72, −2.02]	<0.001	39.2%
Administration method					
Intramuscular injection	4	38/34	−2.82 [−4.11, −1.53]	<0.001	71.3%
Intraperitoneal injection	9	72/24	−10.99 [−14.05, −7.92]	<0.001	67.0%
Intragastric gavage	2	15/15	−2.04 [−3.12, −0.97]	<0.001	18.3%
Follow-up duration					
< 10 days	10	77/29	−10.10 [−13.35, −6.85]	<0.001	80.9%
≥ 10 days	5	48/44	−2.54 [−3.54, −1.54]	<0.001	66.5%

HS, histopathological score; DSS, dextran sodium sulfate; TNBS, 2,4,6-trinitrobenzenesulfonic acid; OMT, oxymatrine; SMD, standard mean difference; CI, confidence interval; E/C, experiment/control; IBD, inflammatory bowel disease.

#### 3.4.3 Colon length

**Figure 4:**
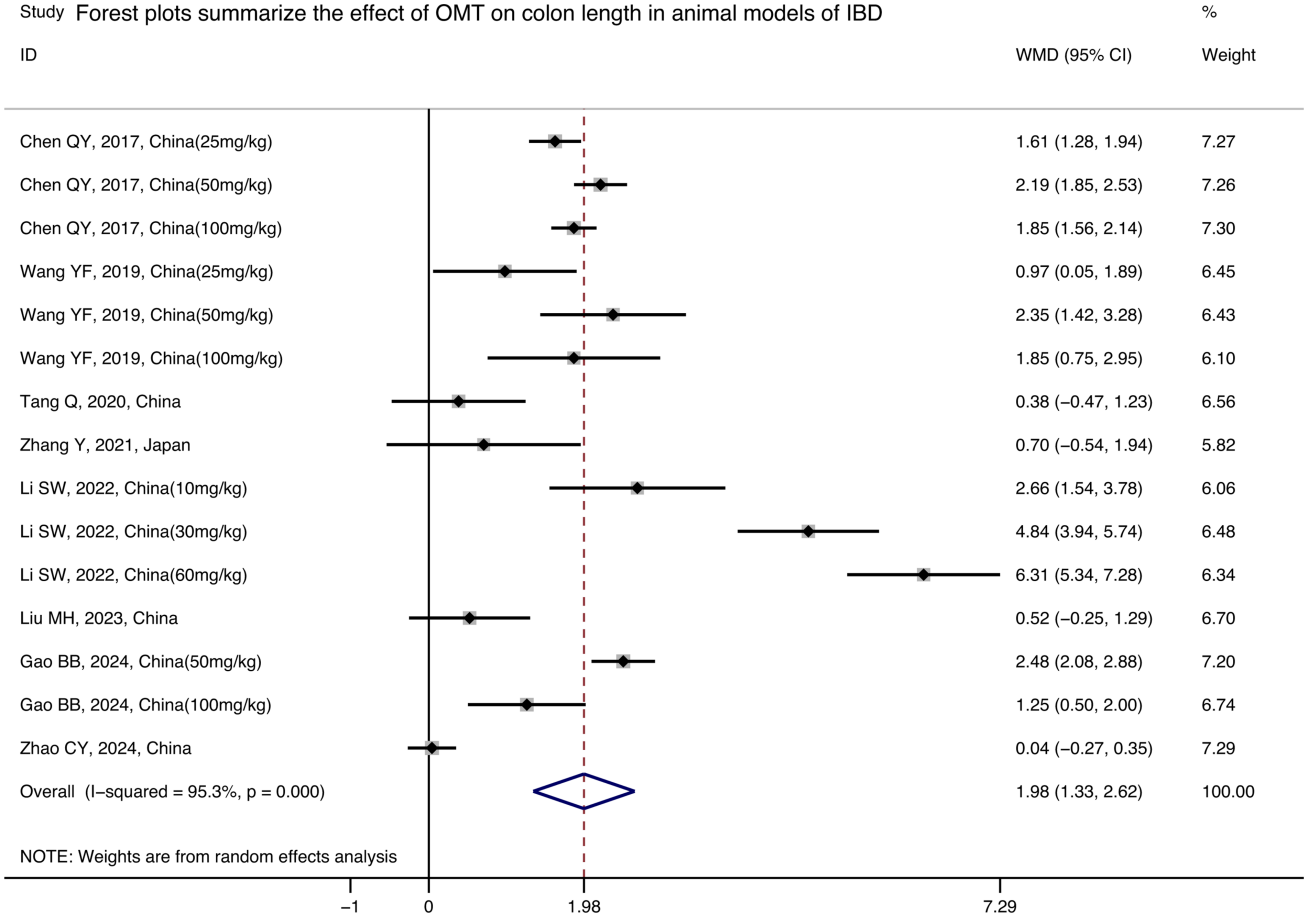
Forest plots summarize the effect of OMT on colon length in animal models of IBD.

Among the included studies, 8 provided colon length (7, 15, 17, 42–46). The final results showed that the colon length of IBD mice receiving OMT intervention was prolonged [*n* = 115/59, WMD = 1.98 (1.33, 2.62), *p* < 0.001; Figure 4].

#### 3.4.4 Inflammation-related indicators

A total of 5 studies measured the level of IL-6 secreted by the colon (17, 42, 43, 46, 47). Following statistical analysis, it was found that OMT can inhibit its secretion level [*n* = 57/33, SMD = −3.77 (−5.47, −2.06), *p* < 0.001; Table 5]. 4 studies reported on the amount of IL-1β in the colon (17, 42, 43, 46), and the OMT intervention was found to significantly reduce the amount of this inflammatory factor [*n* = 49/25, SMD = −7.39 (−11.06, −3.72), *p* < 0.001; Table 5]. 4 studies described the concentration of IL-10 (40, 42, 44, 46), but the results were not statistically significant (*p* = 0.426; Table 5). 5 studies examined the TNF-*α* content in colon tissue (17, 42, 43, 46, 47). After combining the data, it was suggested that the TNF-α expression level in the OMT group was reduced [*n* = 57/33, SMD = −4.54 (−6.18, −2.90), *p* < 0.001; Table 5]. Furthermore, 5 studies monitored NF-κB protein levels (7, 40–42, 47), and the results of the data summary indicated that the expression of this inflammatory transcription factor is suppressed [*n* = 55/35, SMD = −3.59 (−5.21, −1.98), *p* < 0.001; Table 5].

**Table 5:** Data analysis results of IL-6, IL-1β, IL-10, TNF-α, NF-κB, MPO, ZO-1, occluding.

Outcomes	Study	Analyzed (E/C)	SMD (95% CI)	*p*-value
IL-6	5	57/33	−3.77 [−5.47, −2.06]	*p* < 0.001
IL-1β	4	49/25	−7.39 [−11.06, −3.72]	*p* < 0.001
IL-10	4	61/27	1.674 [−2.44, 5.79]	*p* = 0.426
TNF-α	5	57/33	−4.54 [−6.18, −2.90]	*p* < 0.001
NF-κB	5	55/35	−3.59 [−5.21, −1.98]	*p* < 0.001
MPO	3	33/17	−2.47 [−3.91, −1.03]	*p* = 0.001
ZO-1	3	53/21	6.32 [3.88, 8.76]	*p* < 0.001
Occludin	3	53/21	6.44 [3.37, 9.51]	*p* < 0.001

IL-6, interleukin-6; IL-10, interleukin-10; IL-1β, interleukin-1β; TNF-α, tumor necrosis factor-α; NF-κB, nuclear factor-κB; MPO, myeloperoxidase; ZO-1, zonula occludens-1.

#### 3.4.5 Myeloperoxidase

MPO is a marker of neutrophil activation and can be used to assess the level of intestinal inflammation. 3 studies documented MPO activity (43, 44, 46). The combined data showed a substantial decrease in MPO activity following the OMT intervention [*n* = 33/17, SMD = −2.47 (−3.91, −1.03), *p* = 0.001; Table 5]. This result suggested that OMT might effectively reduce the degree of inflammatory response in the body and might positively reduce oxidative stress levels.

#### 3.4.6 Tight junction proteins

As the main tight junction (TJ) proteins, zonula occludens-1(ZO-1)and occludin are important structures for maintaining the integrity of the intestinal barrier (48). 3 studies simultaneously reported the expression levels of ZO-1 and occludin in the colon tissue of IBD mice (42, 44, 46). The combined analysis showed that the expression of ZO-1 [*n* = 53/21, SMD = 6.32 (3.88, 8.76), *p* < 0.001, Table 5] and occludin [*n* = 53/21, SMD = 6.44 (3.37, 9.51), *p* < 0.001, Table 5] in the colon of mice treated with OMT was significantly increased. These results indicate that OMT may protect and repair the intestinal barrier in mice.

#### 3.4.7 Publication bias

**Figure 5:**
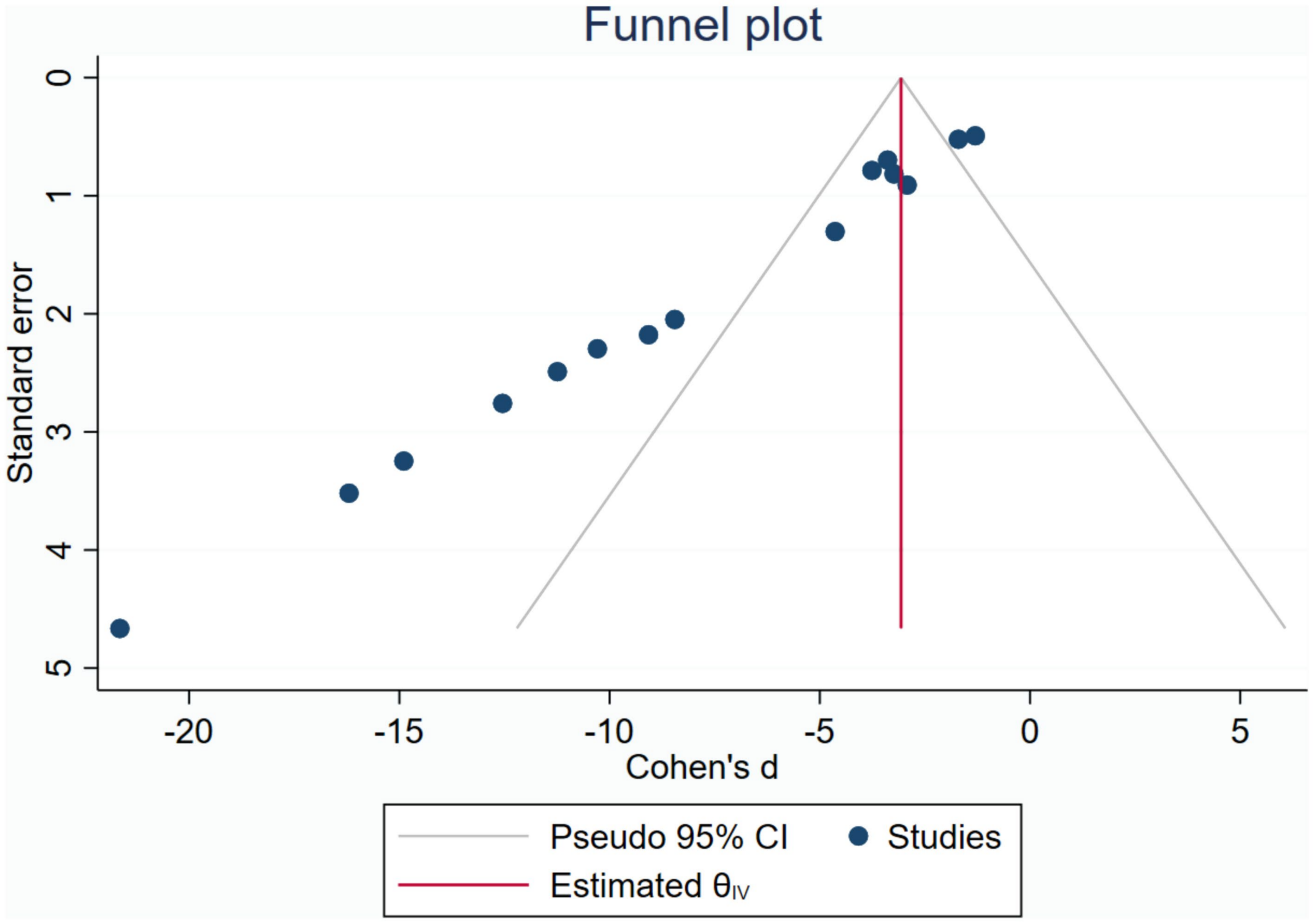
Funnel plot.

**Figure 6:**
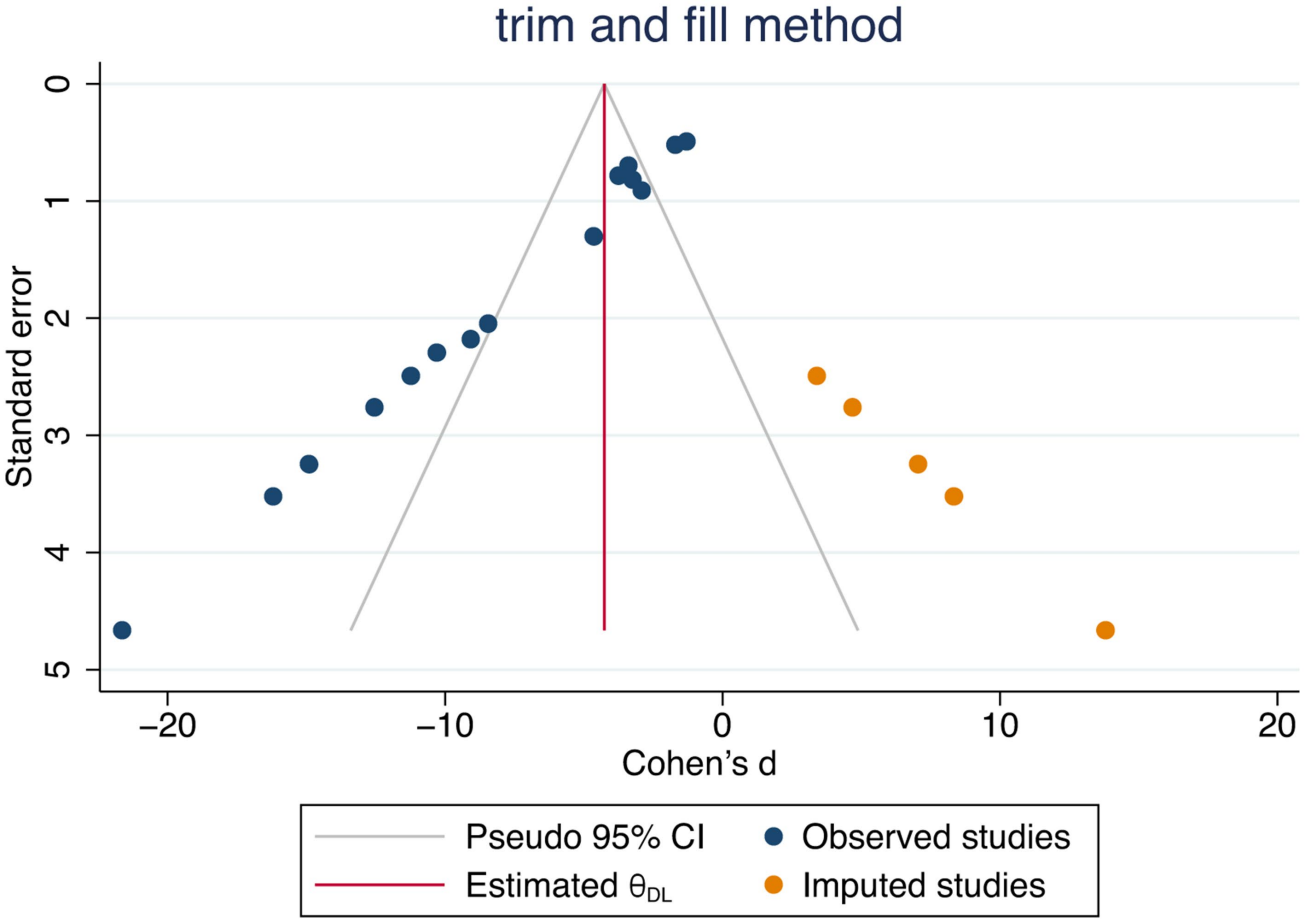
Trimmed and filled funnel plot.

Publication bias was assessed using the funnel plot for indicators HS. The funnel plot for HS (Figure 5) showed significant asymmetry, suggesting possible publication bias. The publication bias may stem from high heterogeneity among studies and language bias (predominantly from a single country). To ensure the stability of our findings, we employed the trim-and-fill test, which confirmed that this publication bias did not compromise the stability of our results (Figure 6).

#### 3.4.8 Sensitivity analysis

The results of meta-analysis have high heterogeneity, and no clear source of heterogeneity has found after subgroup analysis. In order to verify the stability of the results, we analyzed the sensitivity of HS, colon length, TNF-*α*, IL-6, IL-1β, NF-κB, IL-10, MPO, ZO-1 and occluding (Supplementary Figure 3). The results of sensitivity analysis showed that none of the studies had a significant impact on the final effect after the literatures were eliminated in turn.

## 4 Discussion

### 4.1 Evidence summary

In this meta-analysis, 12 preclinical studies were included to evaluate the therapeutic effect of OMT on the IBD animal model. The research used SYRCLE’s risk of bias tool to assess the included studies. Although the overall quality is acceptable, most research did not clearly describe blinding and randomization in the methods section. Meta-analysis of the data showed that OMT can delay disease progression in IBD mouse models. OMT can significantly reduce HS, DAI, and various inflammation-related indicators. At the same time, it can protect the intestinal barrier and prevent colon shortening. We made a subgroup analysis of the main outcome indicators (DAI, HS). The results suggested that the moderate-dose OMT (30-60 mg/kg) showed better therapeutic effect, especially in improving HS. Due to the high heterogeneity of the data, sensitivity analyses were performed for each indicator except DAI. The results showed that excluding each study did not affect the stability of the final results. Publication bias was detected in the funnel plot, but it did not affect the overall findings according to the trim-and-fill analysis. Overall, OMT can reduce intestinal inflammation and histopathological scores in the intestine. Consequently, it may be considered a promising candidate for treating IBD.

### 4.2 Possible protective mechanism of oxymatrine

#### 4.2.1 Possible anti-inflammatory mechanism of oxymatrine

The summary data shows that OMT reduced the levels of IL-6, IL-1*β*, TNF-*α*, and NF-κB in colon tissue. This indicates the key role of NF-κB in inflammation regulation.

IBD is a kind of idiopathic inflammatory bowel disease. A large number of inflammatory cytokines can accumulate in the intestinal mucosa of IBD (49). Nuclear factor-κB (NF-κB) is a classic pro-inflammatory transcription factor. Its level of activation is strongly linked to the level of colonic inflammation activity (50–52). Toll-like receptor (TLR) mediated signaling pathways are also critical in causing colonic inflammation outbreaks (53, 54).

Abnormal gut microbiota in IBD can activate TLR, leading to IκBα (an NF-κB inhibitor) phosphorylation and increased downstream protein NF-κB expression (41, 55, 56). An increase in NF-κB levels can activate T cells to release inflammatory factors like IL-1β, IL-6, and TNF-α. A study showed that OMT can inhibit the TLR/NF-κB pathway by improving gut microbiota (7, 42). OMT can also stimulate β2-adrenergic receptors (β2AR), promoting β-arrestin2 expression and reducing IκBα degradation. Through this way to interrupt the TLR/NF-κB pathway (41). Several studies have shown that OMT can reduce the release of cytokines, adhesion molecules, etc. by reducing the expression level of NF-κB (29, 40, 47).

The phosphatidylinositol 3-kinase (PIK3) / protein kinase B (AKT) signaling pathway is involved in cell survival, differentiation, apoptosis, and autophagy (57, 58). This pathway is active in IBD patients. Activated PIK3 mediates the phosphorylation of AKT, which in turn activates rapamycin target protein (mTOR). mTOR can initiate the NF-κB pathway to cause inflammation (59). Research by Chen has demonstrated that OMT can inhibit the PIK3/AKT pathway to improve colonic inflammation (15).

Ras homolog gene family, member A (RhoA) activity increased in colonic tissues with IBD (60). Rho kinase (ROCK) is a downstream effector of RhoA. NF-κB is an important downstream target of ROCK. Activation of ROCK boosts NF-kB phosphorylation. This impacts Th17 differentiation and the balance between Th17 and Treg cells (61). The series of reactions eventually lead to the body’s immune imbalance and inflammation of intestinal. An animal experiment has demonstrated that OMT can relieve DSS-induced intestinal inflammation by regulating RhoA/ROCK pathway (44).

#### 4.2.2 Possible antioxidant mechanism of oxymatrine

The results of our quantitative analysis were positive for MPO. This suggests that OMT may alleviate intestinal inflammation by anti-oxidative stress.

Oxidant stress refers to the imbalance between antioxidant and oxidative effects. Excessive reactive oxygen metabolites from oxidative stress can damage the structure and function of intestine (62, 63). Myeloperoxidase (MPO) is a member of the heme peroxidase superfamily. It can be found in neutrophils, monocytes, and macrophages. MPO can produce various compounds with pro-oxidant properties. These compounds promote oxidative stress by oxidizing LDL and reducing NO bioavailability (64). Reactive oxygen species (ROS) are the most common free radicals in the human body. Excess ROS in the intestine can trigger an oxidative stress response. This response promotes the production of pro-inflammatory factors and cause intestinal inflammation (65). Zheng has shown that OMT can effectively inhibit MPO activity and reduce ROS formation in IBD mice model (32). Malondialdehyde (MDA) is a lipid peroxidation product. It is a crucial indicator of oxidative damage (66). Superoxide dismutase (SOD) is an endogenous antioxidant enzyme in the human body. SOD can catalyze the conversion of superoxide free radicals to hydroperoxides (67). Glutathione (GSH) can mitigate the damage to the body caused by free radicals and electrophilic chemicals (68). Previous studies have confirmed that OMT can reduce MPO and MDA levels and increase SOD and GSH levels to regulate oxidative stress in colitis mice (43, 44, 46). It provides strong evidence for the antioxidant effect of OMT in IBD.

#### 4.2.3 Protective effect of oxymatrine on intestinal barrier

An intact intestinal barrier can reduce the entry of harmful substances into the body and prevent the production of inflammation (69, 70). Therefore, maintaining the integrity of the intestinal barrier is of great significance for IBD patients. Tight junction (TJ) proteins ensure tight junctions between intestinal epithelial cells to regulate intestinal barrier permeability (71). It is composed of transmembrane proteins, scaffold proteins and cytoskeleton. ZO-1 is one of the most important scaffold proteins. It interacts with transmembrane proteins through the PDZ domain, while connecting the cytoskeleton to maintain the integrity of the TJ structure (72). ZO-1 dysfunction can lead to changes in tight junction function, which is often used as a marker to assess the barrier function and permeability of various tissues (73). Previous studies have shown that ZO-1-deficient mouse epithelial cells exhibit protein loss leading to delayed junction assembly and intestinal barrier dysfunction (74). Occludin is a transmembrane protein in tight junctions and plays an important role in the regulation of the intestinal barrier. Decreased Occludin expression can cause increased barrier permeability and chronic inflammation, suggesting a correlation with the progression of IBD (71). The results of the included studies showed that the expression levels of ZO-1 and occludin were significantly increased after OMT intervention (42, 44, 46). This suggests that OMT may have a protective effect on the intestinal barrier. However, some studies have found that TNF-*α* can induce the decomposition of tight junctions (ZO-1, occludin, etc.) and lead to barrier loss (75). This also points out that OMT may improve the intestinal barrier through its anti-inflammatory effect.

### 4.3 Appropriate dosage and mode of oxymatrine administration

To use oxymatrine more rationally, we conducted a subgroup analysis of the dose, administration method and administration time of OMT. These findings indicated that the moderate-dose OMT (30-60 mg/kg) showed the best therapeutic effect. In particular, it improved HS far beyond the low and high dose groups. Five studies showed that OMT had comparable therapeutic effects to mesalazine (7, 16, 17, 40, 41). However, there are relatively few studies on using moderate-dose OMT. We still need more research to support this assertion, and increase the safety research of drugs to provide evidence for clinical use. Regarding the method of administration, intraperitoneal injection has a better effect on improving HS than intragastric gavage and intramuscular injection. Intraperitoneal injection makes the drug act more directly on the intestines to provide a higher concentration at the lesion. It may enhance drug efficacy in the intestines by minimizing metabolism and first-pass effect. Generally, administration of OMT at 30-60 mg/kg by intraperitoneal injection is recommended.

There is significant variation in the administration methods and doses of OMT, which could be confounding factors contributing to the high heterogeneity of results. By integrating the data, we found that the dose of OMT administered via intramuscular injection was consistently 63 mg/kg, while the dose for intraperitoneal injection varied between 10 and 100 mg/kg due to different group allocations. The doses for oral gavage were higher, at 100 mg/kg and 180 mg/kg. The reasons for these variations could be: (1) some studies did not perform dose grouping; (2) different research teams had diverse foundational research, leading to variations in dose selection; (3) different administration methods result in variations in drug absorption rates and bioavailability, such as the higher doses required for oral gavage, possibly due to slower absorption and lower bioavailability compared to the other two methods. Overall, further exploration is needed regarding the dose and administration methods of OMT. Studies on different administration methods and doses will help identify the optimal drug application range, providing sufficient evidence for the rational use of OMT.

### 4.4 Advantages and limitations of this review

This review should be the first systematic review and meta-analysis to report the effects of oxymatrine on animal models of IBD. Evidence regarding the clinical efficacy of oxymatrine in patients with IBD remains limited. The existing studies have not discussed the relationship between the dose and efficacy of OMT in the treatment of IBD. In this meta-analysis, we have preliminarily evaluated the optimal dose, administration method and administration time of OMT through multi-dimensional subgroup analysis. Through the analysis of a variety of outcome indicators, this study elaborates the possible mechanism of OMT in the treatment of IBD. These data provide more evidence-based support for the clinical application of oxymatrine preparations. However, there are some limitations in this meta-analysis: (1) The limited quantity of studies searched and included resulted in some indicators being unable to be analyzed due to insufficient data. (2) Some of the data was obtained using data extraction tools rather than raw data, which may impact the conclusions. (3) There was significant heterogeneity in the results. Even after subgroup analysis, no apparent source of heterogeneity was found. This may be due to differences in the specific implementation of modeling, the animal-rearing environment, or the sample extraction method. (4) It is still unclear whether there are differences in the efficacy of OMT in different doses for IBD. This issue might be linked to the limited number of included studies. Additional research is needed to provide more comprehensive data.

### 4.5 Future research direction

Although oxymatrine has a significant effect on IBD mice, it is still challenging to apply it in clinic. The sample size of the current study is still too small, and more experiments with large samples are needed to support our study. It is necessary to add different dose groups in the experiment to better calculate the dose-effect relationship. Oxymatrine has been used in the clinical treatment of hepatitis B, liver fibrosis and skin diseases, without obvious liver and kidney damage and adverse reactions (76). However, some studies have shown that it can cause obvious toxicity when used in large doses, and more experiments are still needed to confirm its safety (77). Although the use of oxymatrine injection is recommended in this study, more efficient and convenient dosage forms need to be developed for clinical use. Large-scale multi-center clinical trials can be conducted if safety permits. In general, oxymatrine is a potential drug for the treatment of IBD due to its low price and few side effects.

## 5 Conclusion

Our meta-analysis showed that oxymatrine can significantly control the development of the disease in an animal model of IBD. Quantitative analysis of DAI, histopathology, various inflammatory indicators, and MPO and TJ showed that oxymatrine exerts an antioxidant and anti-inflammatory effect in IBD mice, thereby reducing intestinal inflammation, strengthening barrier protection, and regulating intestinal flora, aiming to improve IBD symptoms. For reference, it is recommended to choose oxymatrine at 30–60 mg/kg as much as possible, as its effectiveness and safety are better guaranteed. In addition, intraperitoneal injection may be a more appropriate choice. However, there are still some limitations in our research. Further preclinical and even clinical studies are required to confirm the efficacy and safety of oxymatrine.

## Data Availability

The original contributions presented in the study are included in the article/Supplementary material, further inquiries can be directed to the corresponding authors.
