# Supplementary material for: Oxymatrine for inflammatory bowel disease in preclinical studies: a systematic review and meta-analysis
Source: Front Med (Lausanne). 2025 Apr 30;12:1542953. doi: 10.3389/fmed.2025.1542953 (PMC12075229; doi:10.3389/fmed.2025.1542953)
Supplement: Supplementary file 1 [file Table_1.docx]

Supplementary Material

**sTable 1 SYRCLE’s tool for assessing risk of bias (Hooijmans et al., 2014)**

| Item | Assessment methods |
| --- | --- |
| Sequence generation | Did the researcher describe the specific randomization method? Including random number table method/computer generated method, etc. |
| Baseline characteristics | Were the groups similar at baseline or were they adjusted for confounders in the analysis? |
| Allocation concealment | Were the trial groups randomly coded by a third party to achieve unpredictability of the random sequence? |
| Random housing | Were animals randomly housed during the experiment? |
| Blinding (study team) | Were all the experimental unaware of which intervention each animal received? |
| Random outcome assessment | Were animals randomly selected for outcome assessment? |
| Blinding  (outcome assessors) | Was the outcome assessor blinded? To blind outcome assessors from knowing which intervention each animal received. |
| Incomplete outcome data | Were the experimental data fully reported in the article? If data were missing, are the reasons explained? |
| Selective outcome reporting | Were reports of the study free of selective outcome reporting? |
| Other bias | Was the study apparently free of other problems that could result in high risk of bias? |

Hooijmans, C.R., Rovers, M.M., de Vries, R.B., Leenaars, M., Ritskes-Hoitinga, M., and Langendam, M.W. (2014). SYRCLE's risk of bias tool for animal studies. *BMC Med Res Methodol* 14**,** 43. doi: 10.1186/1471-2288-14-43.
