# Supplementary material for: Oxymatrine for inflammatory bowel disease in preclinical studies: a systematic review and meta-analysis
Source: Front Med (Lausanne). 2025 Apr 30;12:1542953. doi: 10.3389/fmed.2025.1542953 (PMC12075229; doi:10.3389/fmed.2025.1542953)
Supplement: Supplementary file 2 [file Supplementary_file_1.docx]

Supplementary Material

# Retrieval strategy

**PUBMED:**

(((((((("Inflammatory Bowel Diseases"[Mesh]) OR (((Inflammatory Bowel Diseases[Title/Abstract]) OR (Inflammatory Bowel Disease[Title/Abstract])) OR (IBD[Title/Abstract]))) OR ("Colitis, Ulcerative"[Mesh])) OR ((Colitis, Ulcerative[Title/Abstract]) OR (Ulcerative Colitis[Title/Abstract]))) OR ("Crohn Disease"[Mesh])) OR ((((Crohn Disease[Title/Abstract]) OR (Crohn's Disease[Title/Abstract])) OR (Crohns Disease[Title/Abstract])) OR (Crohn's Enteritis[Title/Abstract]))) OR ("Colitis"[Mesh])) OR (Colitis[Title/Abstract])) AND (("oxymatrine" [Supplementary Concept]) OR (((((oxymatrine[Title/Abstract]) OR (kurorinone[Title/Abstract])) OR (kushensu[Title/Abstract])) OR (kushenin[Title/Abstract])) OR (OMT[Title/Abstract])))

**Embase:**

#1. inflammatory AND ('bowel'/exp OR bowel) AND ('diseases'/exp OR diseases)

#2. 'inflammatory bowel diseases':ab,ti OR 'inflammatory bowel disease':ab,ti OR 'ibd':ab,ti

#3. colitis, AND ulcerative

#4. 'colitis, ulcerative':ab,ti OR 'ulcerative colitis':ab,ti

#5. crohn AND disease

#6. 'crohn disease':ab,ti OR 'crohns disease':ab,ti OR 'crohns enteritis':ab,ti

#7. colitis

#8. 'colitis':ab,ti

#9. #1 OR #2 OR #3 OR #4 OR #5 OR #6 OR #7 OR #8

#10. Oxymatrine

#11. 'oxymatrine':ab,ti OR 'kurorinone':ab,ti OR 'kushensu':ab,ti OR 'kushenin':ab,ti OR 'omt':ab,ti

#12. #10 OR #11

#13. #9 AND #12

**Web of science:**

#1. TS = (Inflammatory Bowel Diseases OR Inflammatory Bowel Disease OR IBD OR Colitis, Ulcerative OR Ulcerative Colitis OR Crohn Disease OR Crohn's Disease OR Crohns Disease OR Crohn's Enteritis OR Colitis)

#2. TS = (oxymatrine OR kurorinone OR kushensu OR kushenin OR OMT)

#3. #1 AND #2

**Cochrane library:**

#1. Inflammatory Bowel Diseases OR (Inflammatory Bowel Diseases):ab,ti,kw OR (Inflammatory Bowel Disease):ab,ti,kw OR (IBD):ab,ti,kw OR Colitis, Ulcerative OR (Colitis, Ulcerative):ab,ti,kw OR (Ulcerative Colitis):ab,ti,kw OR Crohn Disease OR (Crohn Disease):ab,ti,kw OR (Crohn's Disease):ab,ti,kw OR (Crohns Disease):ab,ti,kw OR (Crohn's Enteritis):ab,ti,kw OR Colitis OR (Colitis):ab,ti,kw

#2. Oxymatrine OR (oxymatrine):ab,ti,kw OR (kurorinone):ab,ti,kw OR (kushensu):ab,ti,kw OR (kushenin):ab,ti,kw OR (OMT):ab,ti,kw

#3. #1 AND #2

**Medline:**

#1. (MH=(Inflammatory Bowel Diseases)) OR ((((((TI=(Inflammatory Bowel Diseases)) OR AB=(Inflammatory Bowel Diseases)) OR TI=(Inflammatory Bowel Disease)) OR AB=(Inflammatory Bowel Disease)) OR AB=(IBD)) OR TI=(IBD)) OR (MH=(Colitis, Ulcerative)) OR ((((TI=(Colitis, Ulcerative)) OR AB=(Colitis, Ulcerative)) OR AB=(Ulcerative Colitis)) OR TI=(Ulcerative Colitis)) OR (MH=(Crohn Disease)) OR ((((((((TI=(Crohn Disease)) OR AB=(Crohn Disease)) OR AB=(Crohn's Disease)) OR TI=(Crohn's Disease)) OR TI=(Crohns Disease)) OR AB=(Crohns Disease)) OR AB=(Crohn's Enteritis)) OR TI=(Crohn's Enteritis)) OR (MH=(Colitis)) OR ((TI=(Colitis)) OR AB=(Colitis))

#2. (MH=(oxymatrine)) OR ((((((((((TI=(oxymatrine)) OR AB=(oxymatrine)) OR AB=(kurorinone)) OR TI=(kurorinone)) OR TI=(kushensu)) OR AB=(kushensu)) OR AB=(kushenin)) OR TI=(kushenin)) OR TI=(OMT)) OR AB=(OMT))

#3. #1 AND #2
