# Supplementary material for: Oxymatrine for inflammatory bowel disease in preclinical studies: a systematic review and meta-analysis
Source: Front Med (Lausanne). 2025 Apr 30;12:1542953. doi: 10.3389/fmed.2025.1542953 (PMC12075229; doi:10.3389/fmed.2025.1542953)
Supplement: Supplementary file 3 [file Supplementary_file_2.docx]

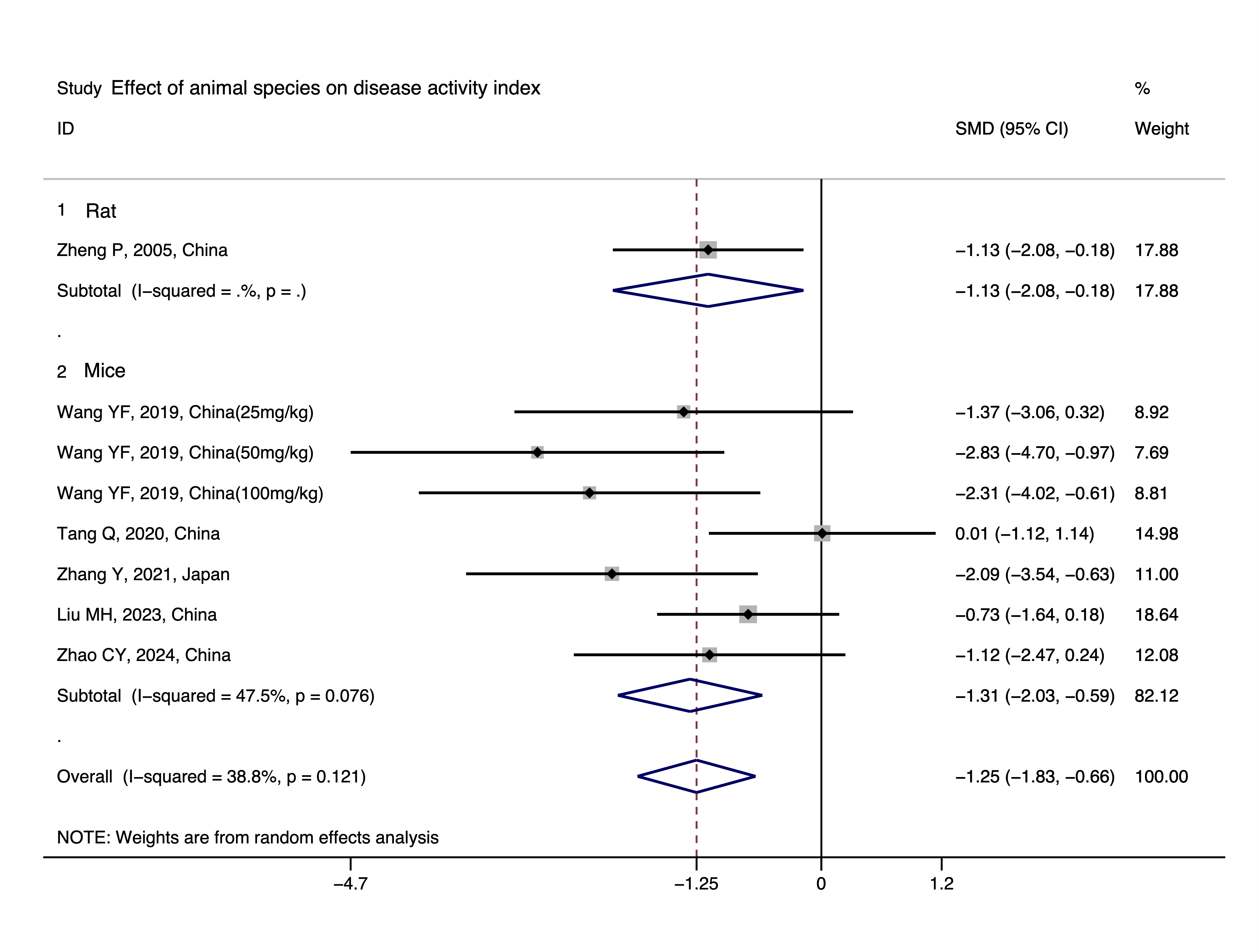
A


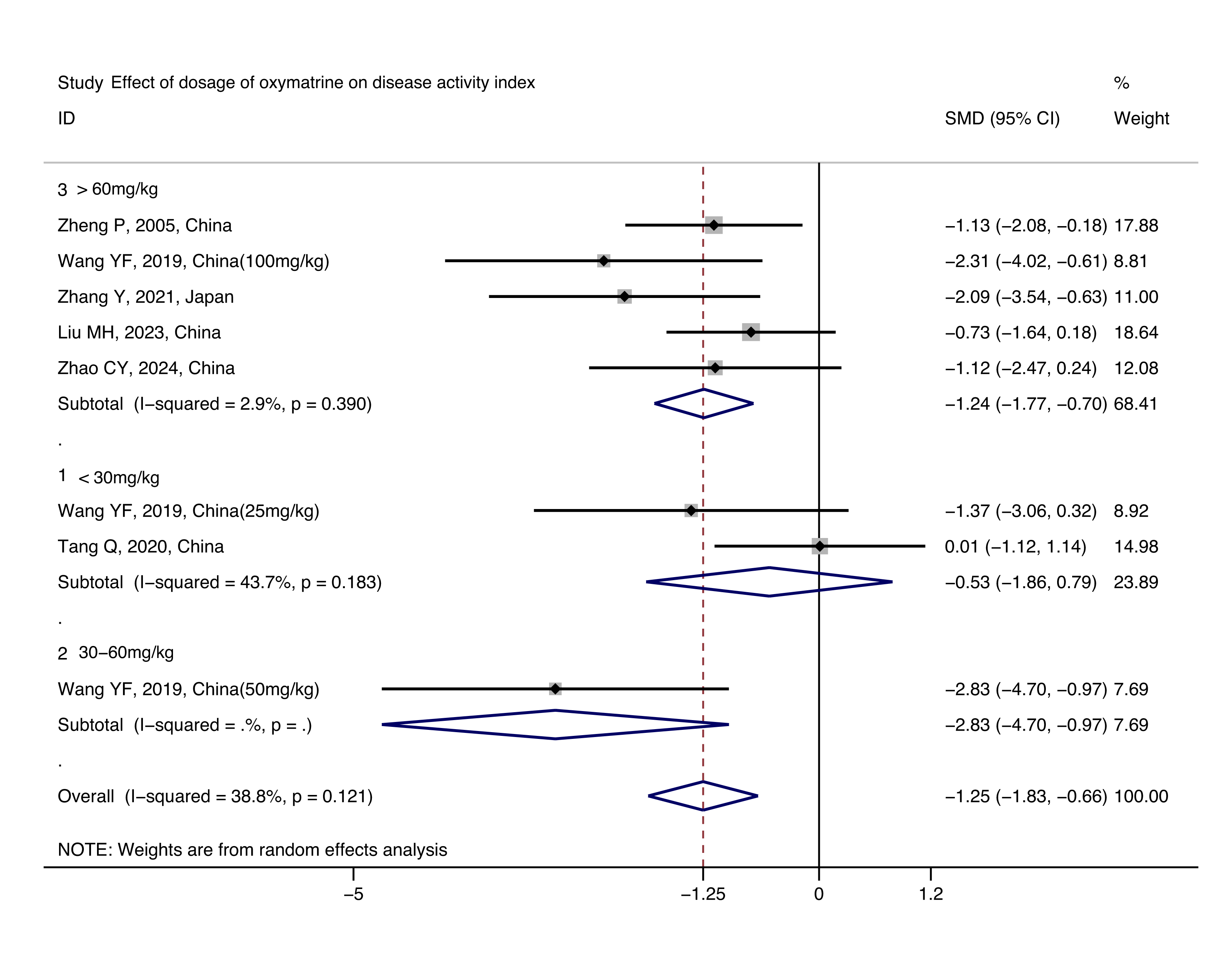
B

**
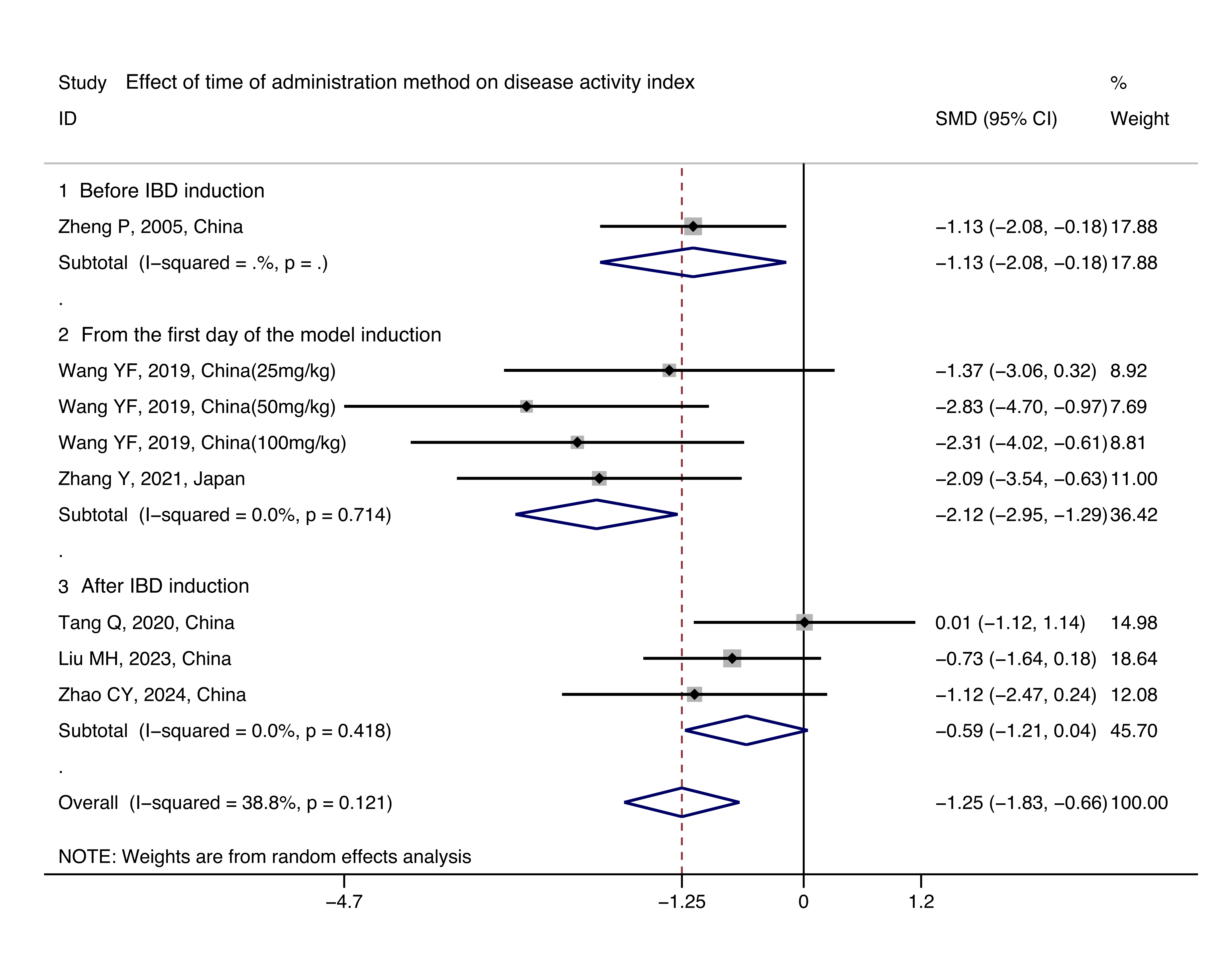
**C


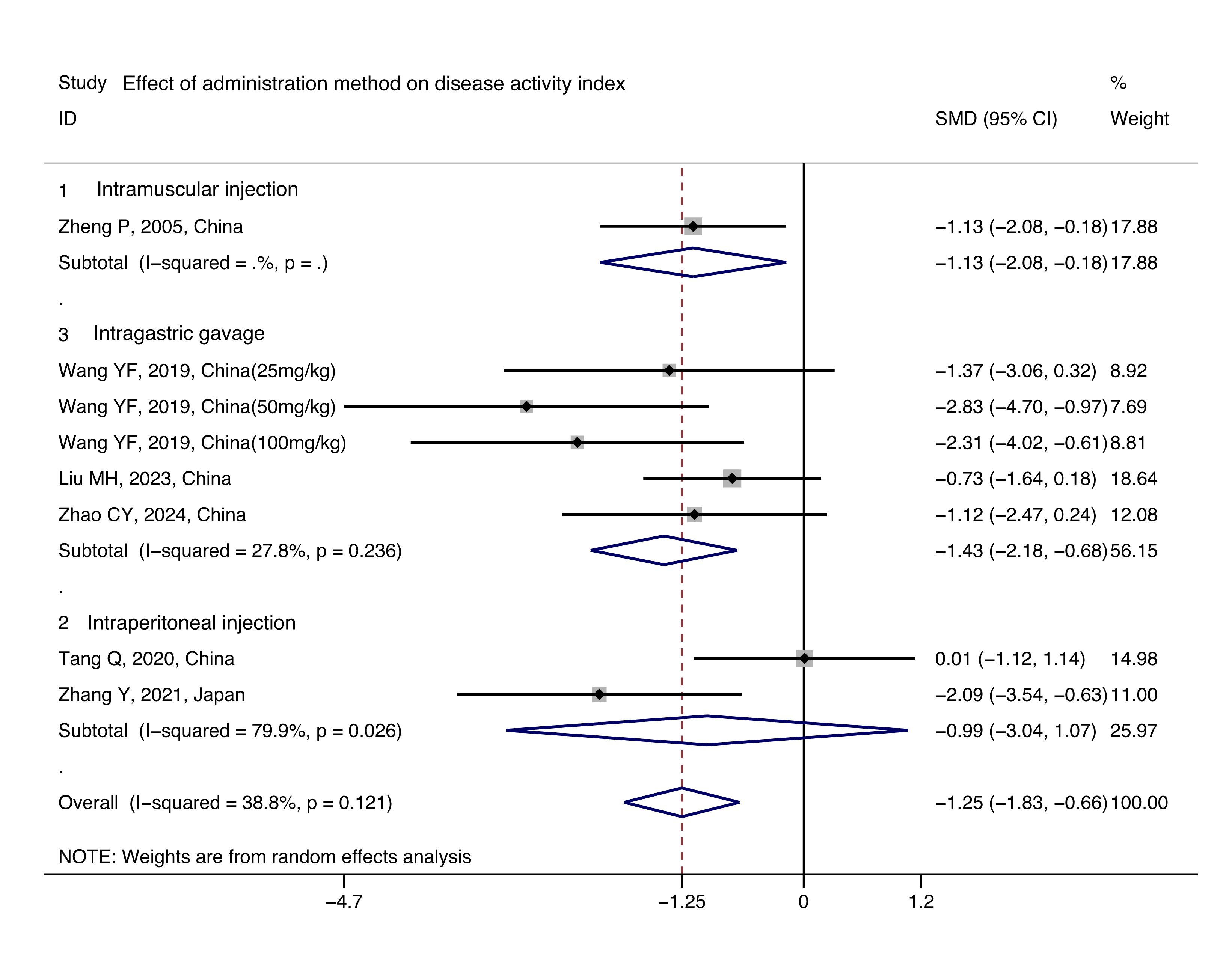
D


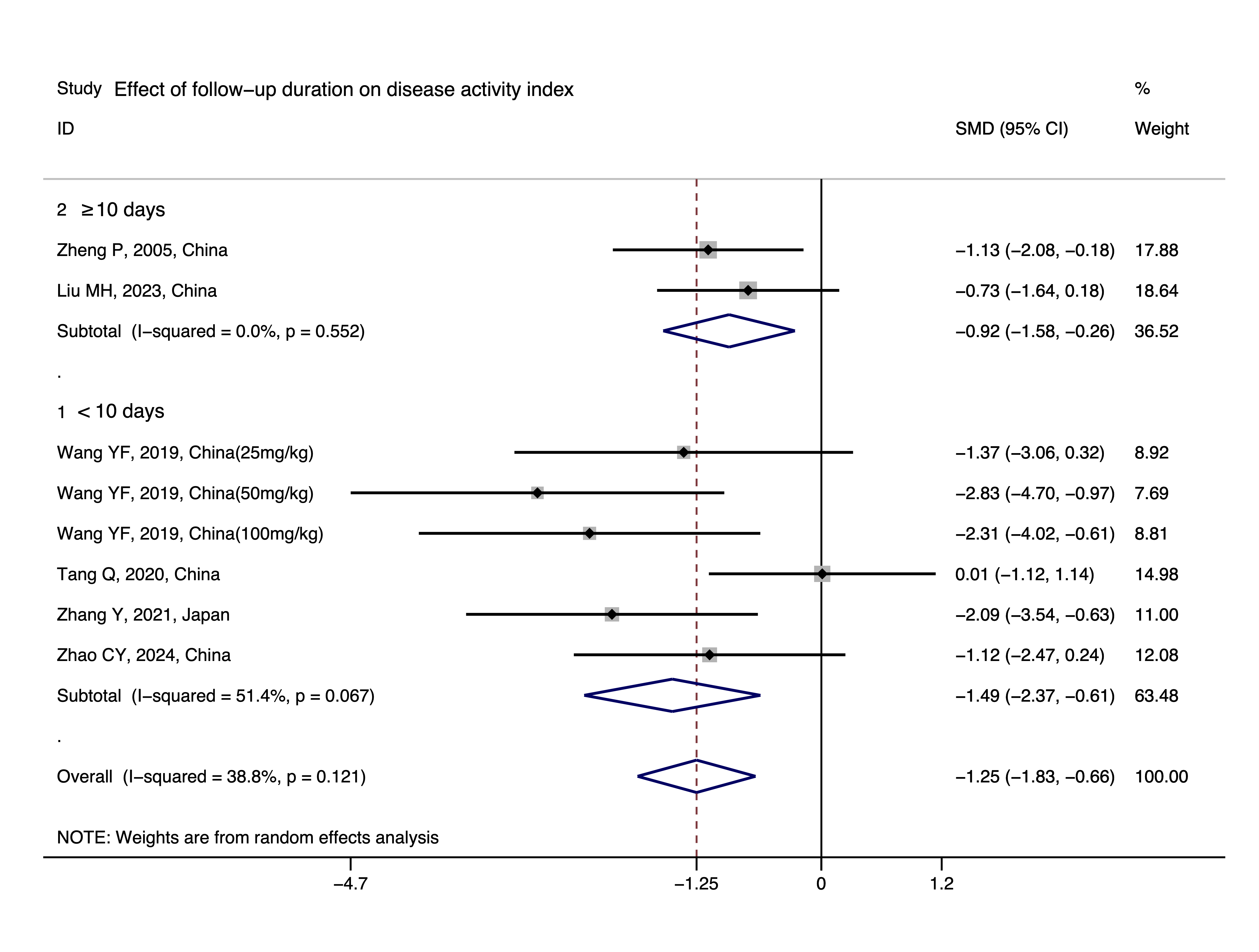
**E**

**Supplementary Figure 1.** Subgroup analysis of disease activity index. **(A)** Effect of animal species on disease activity index; **(B)** Effect of dosage of oxymatrine on disease activity index; **(C)** Effect of time of administration on disease activity index; **(D)** Effect of administration method on disease activity index; **(E)** Effect of follow-up duration on disease activity index.


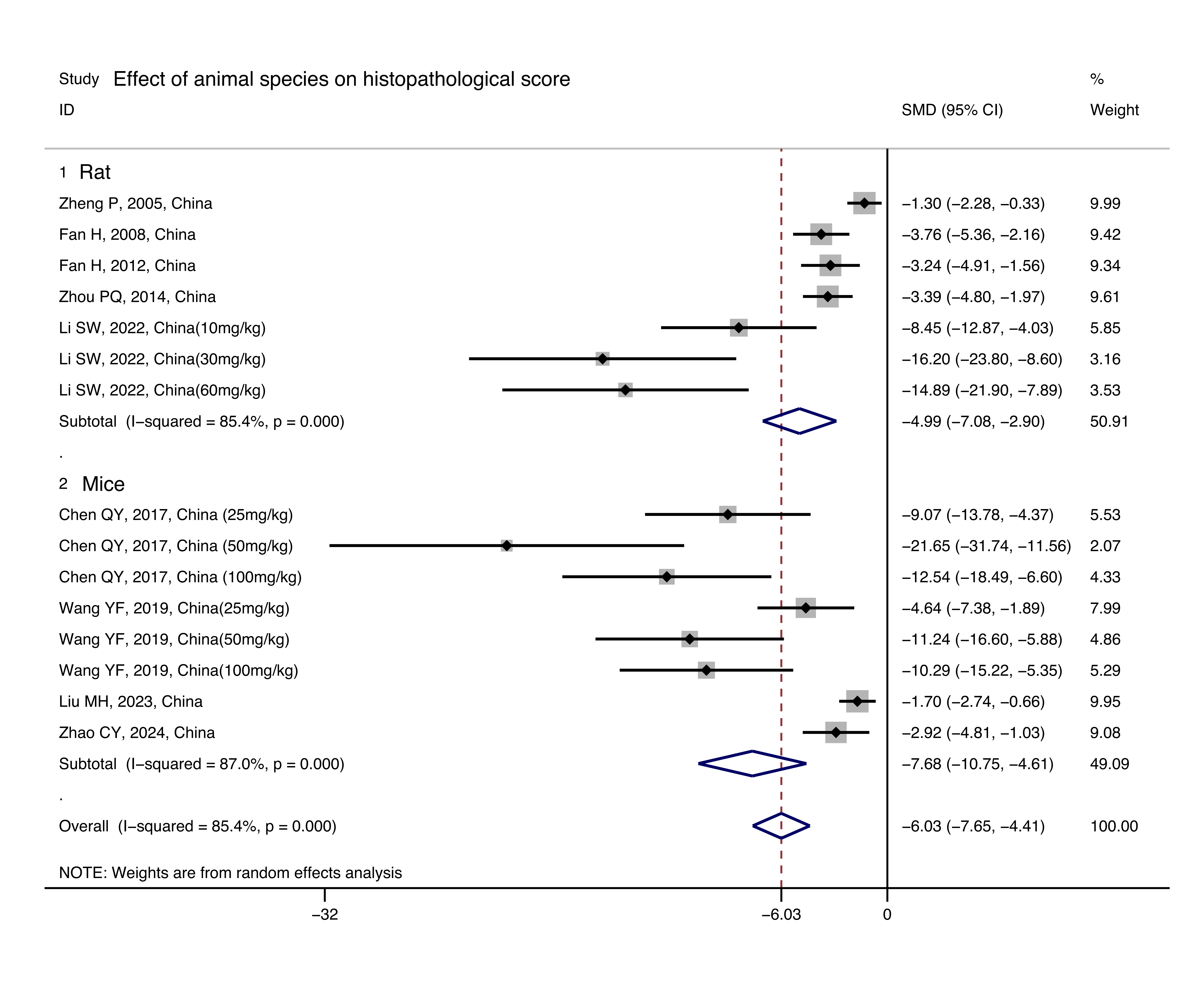
A


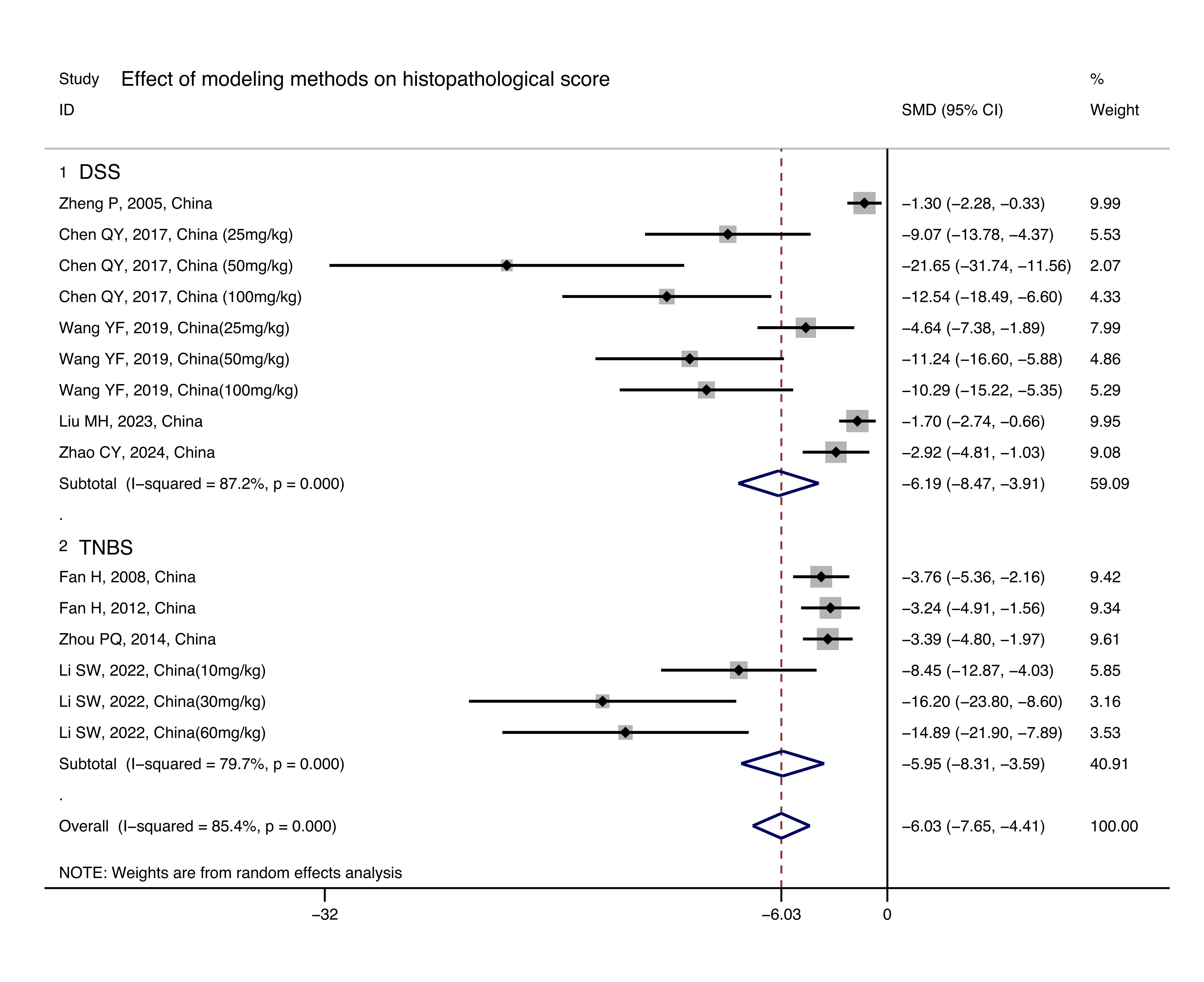
B


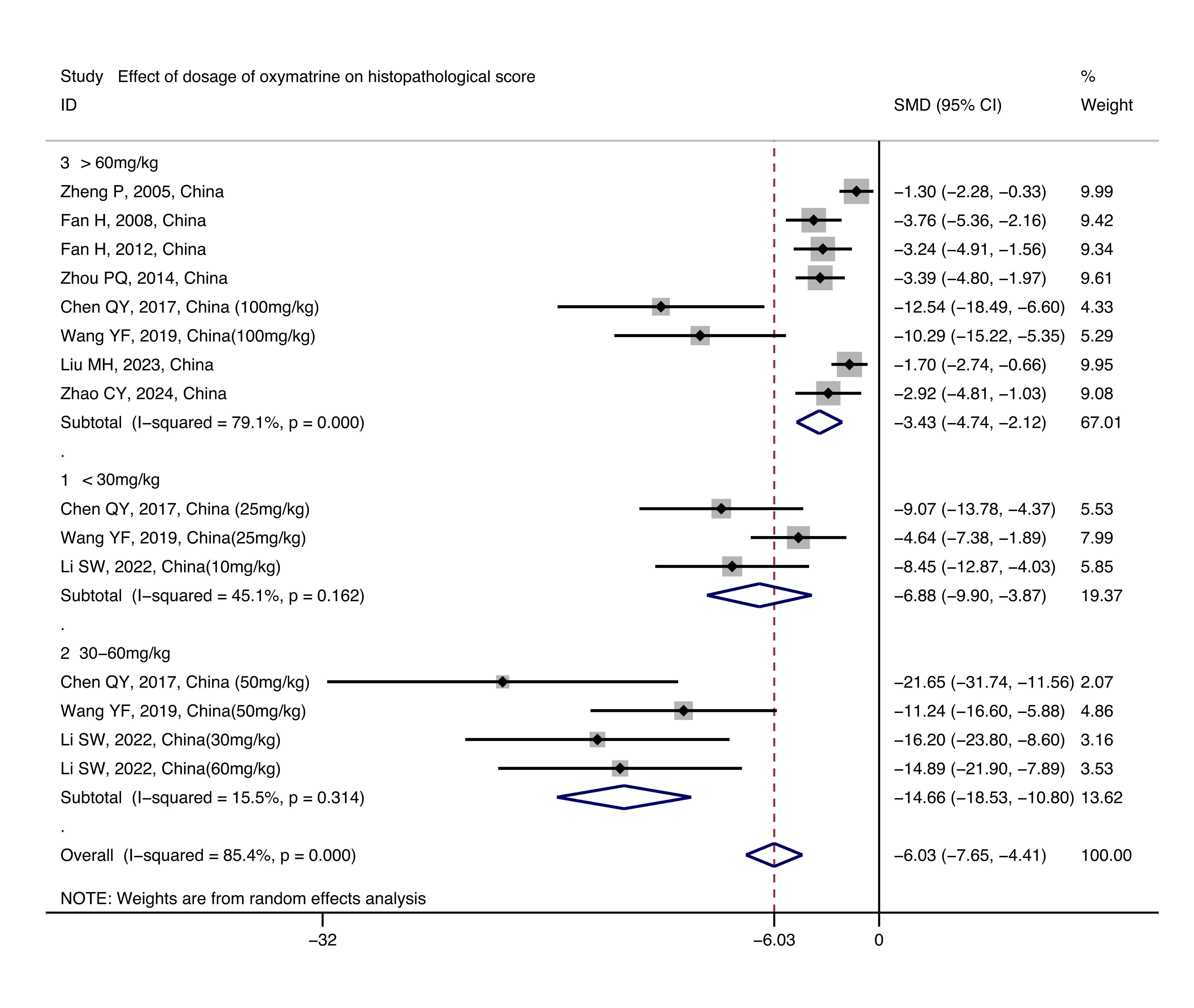
C


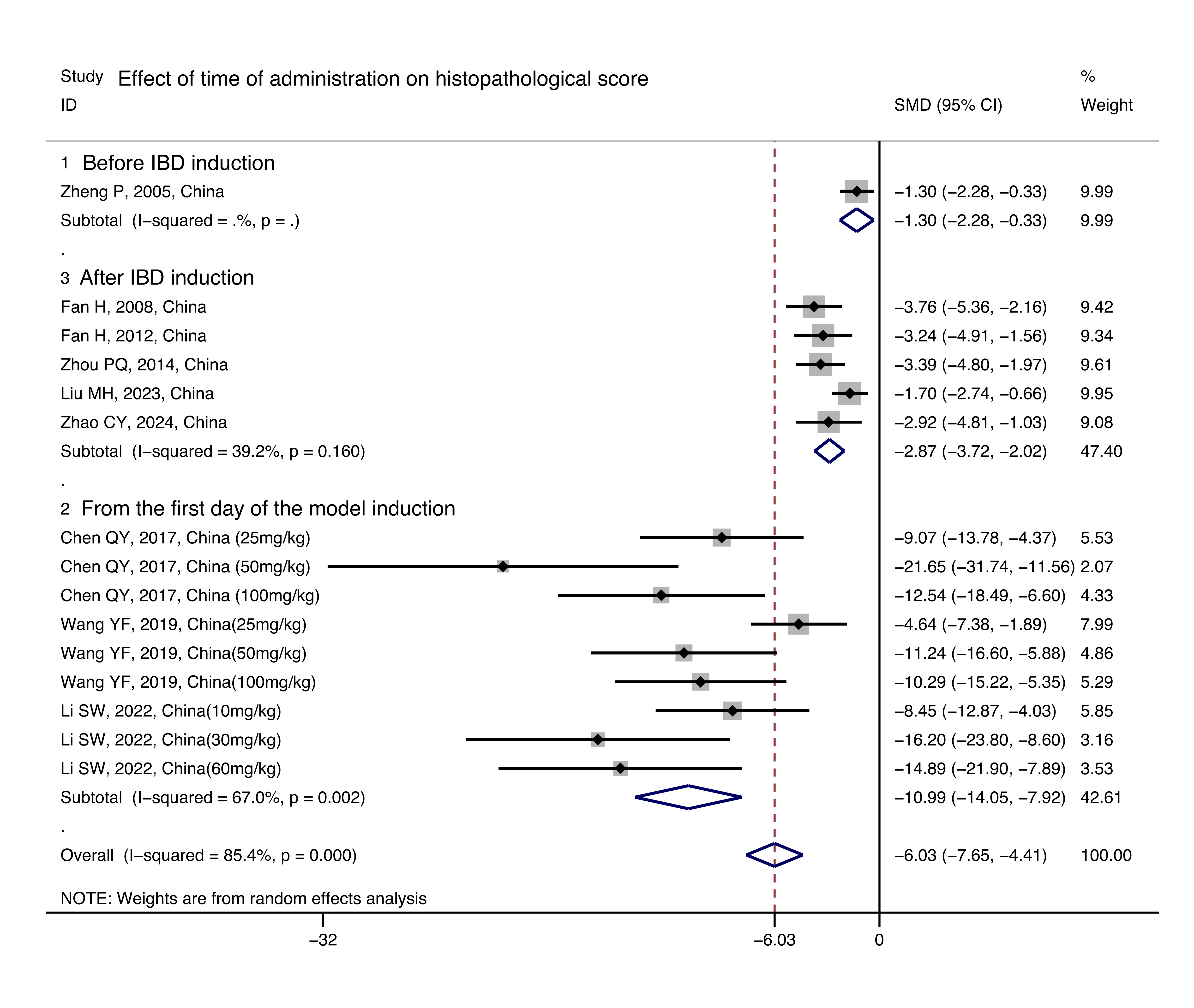
D


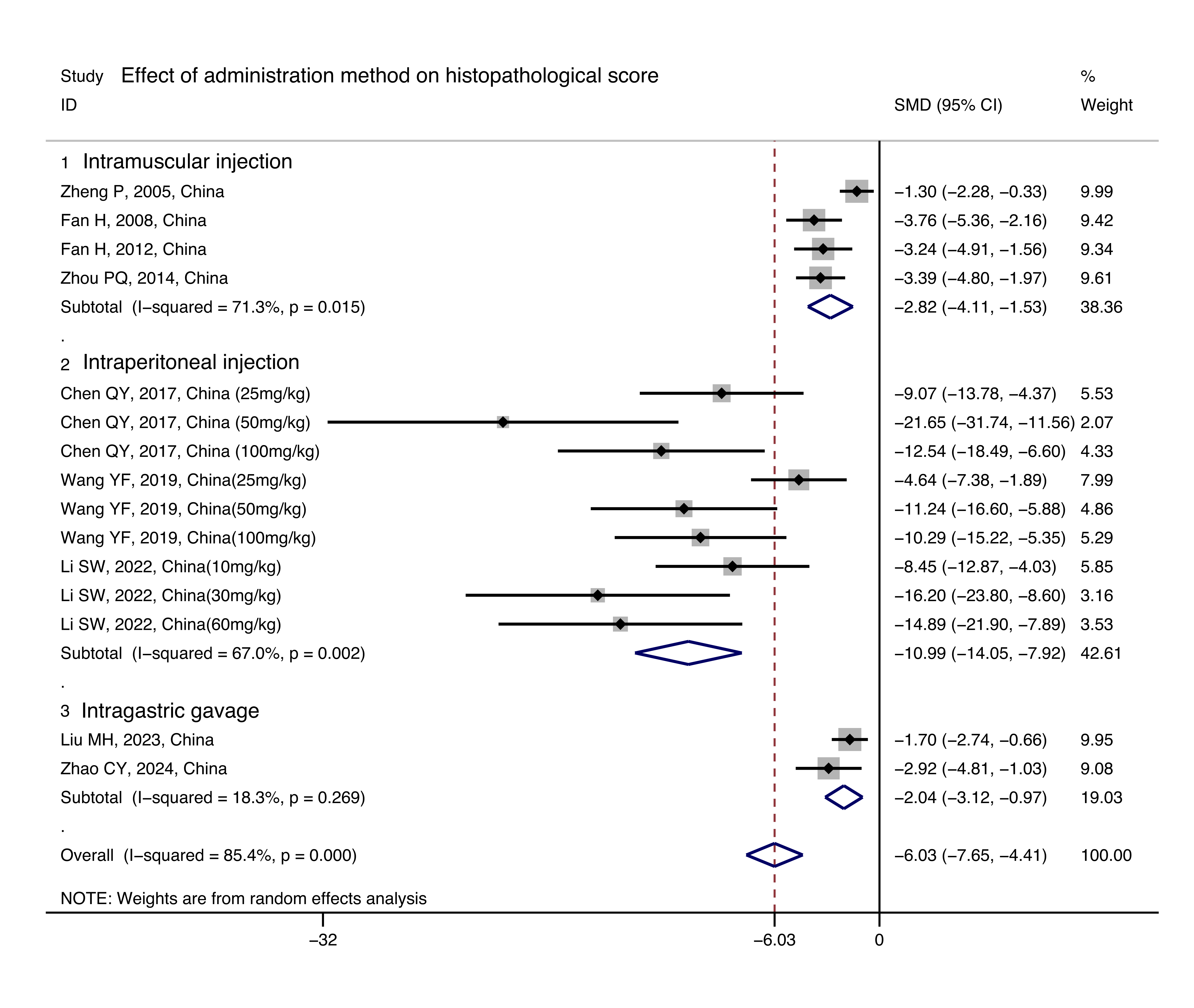
E


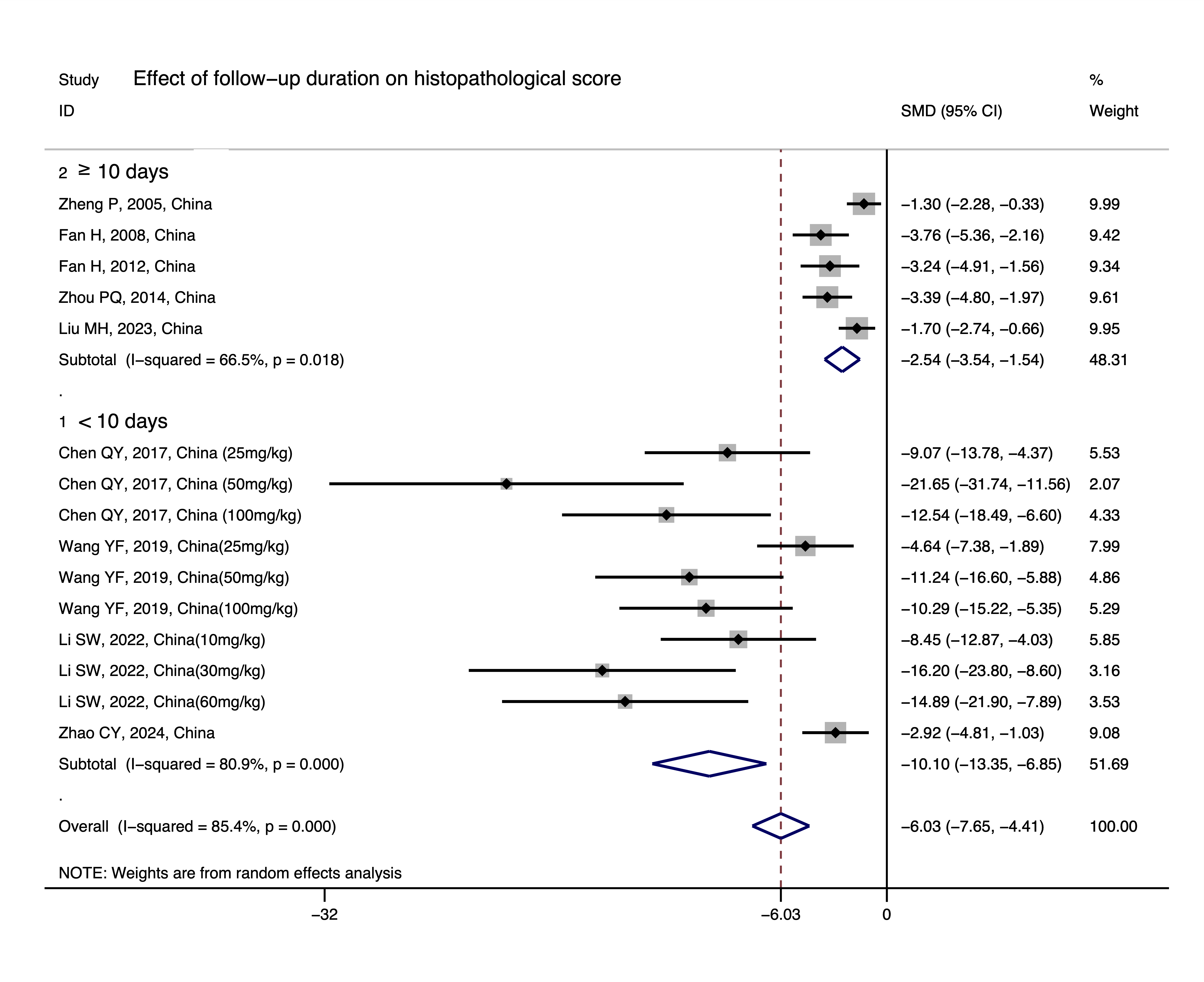
F

**Supplementary Figure 2.** Subgroup analysis of histopathological index. **(A)** Effect of animal species on histopathological index; **(B)** Effect of modeling methods on histopathological index; **(C)** Effect of dosage of oxymatrine on histopathological index; **(D)** Effect of time of administration on histopathological index; **(E)** Effect of administration method on histopathological index; **(F)** Effect of follow-up duration on histopathological index.

A


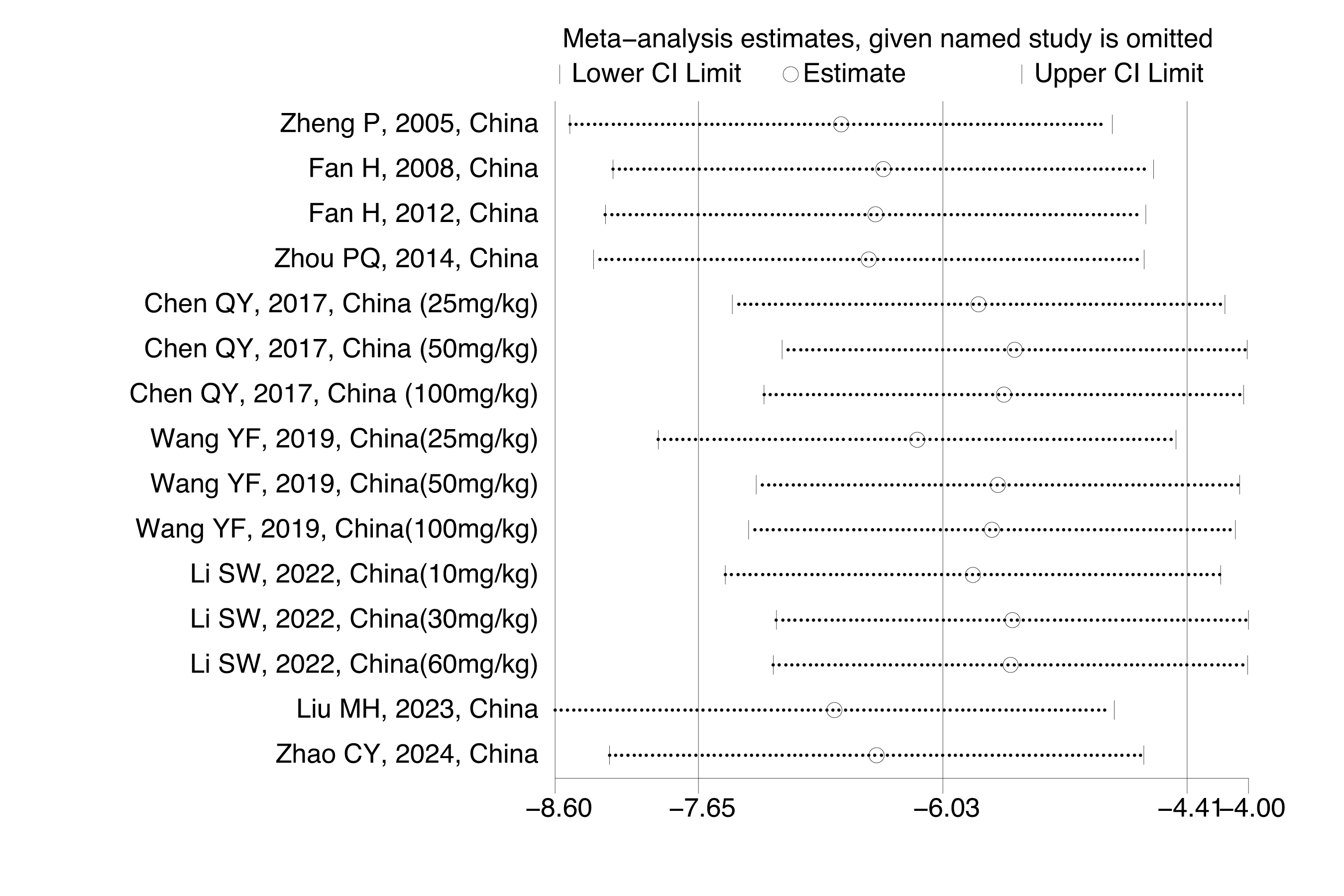


B


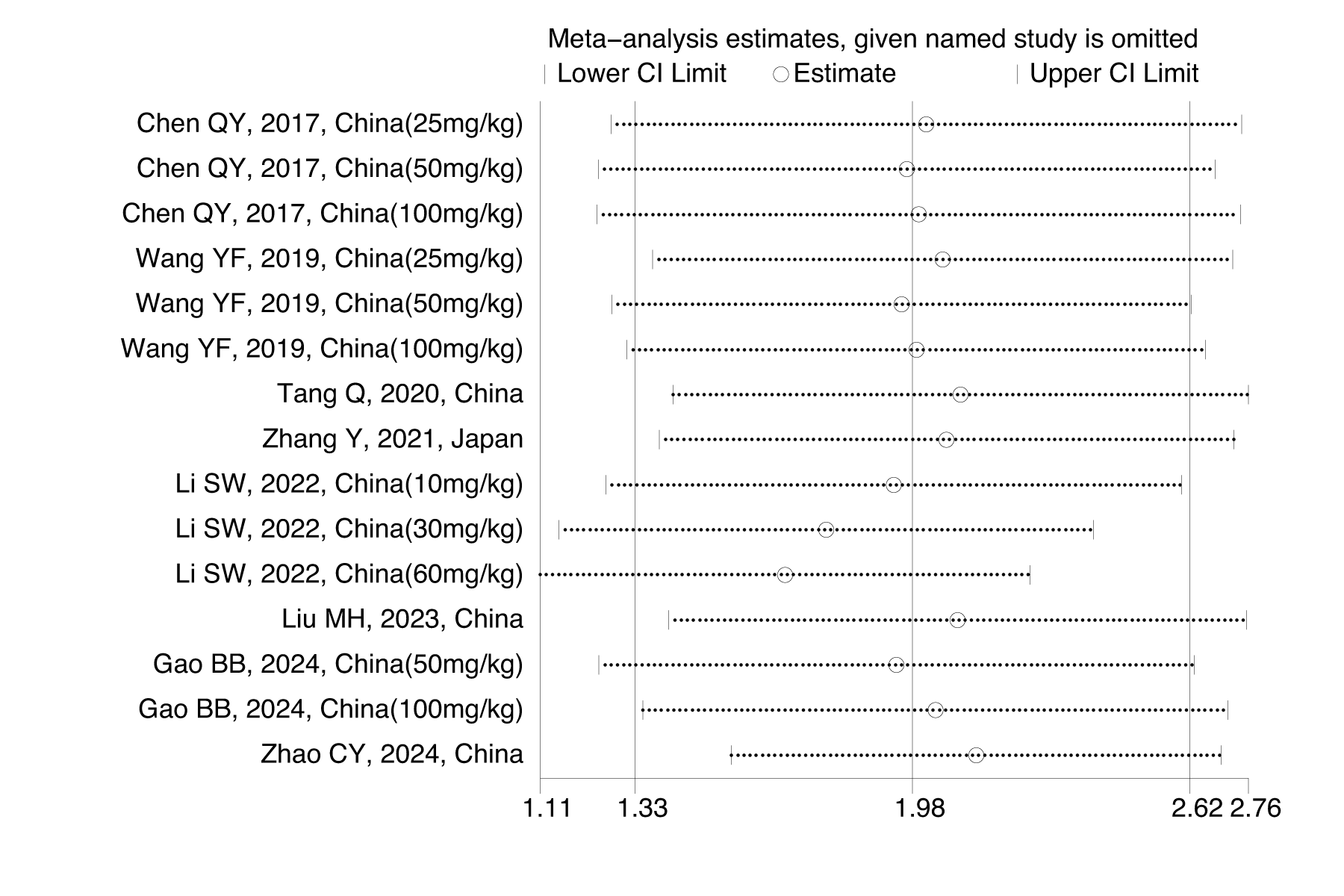


C


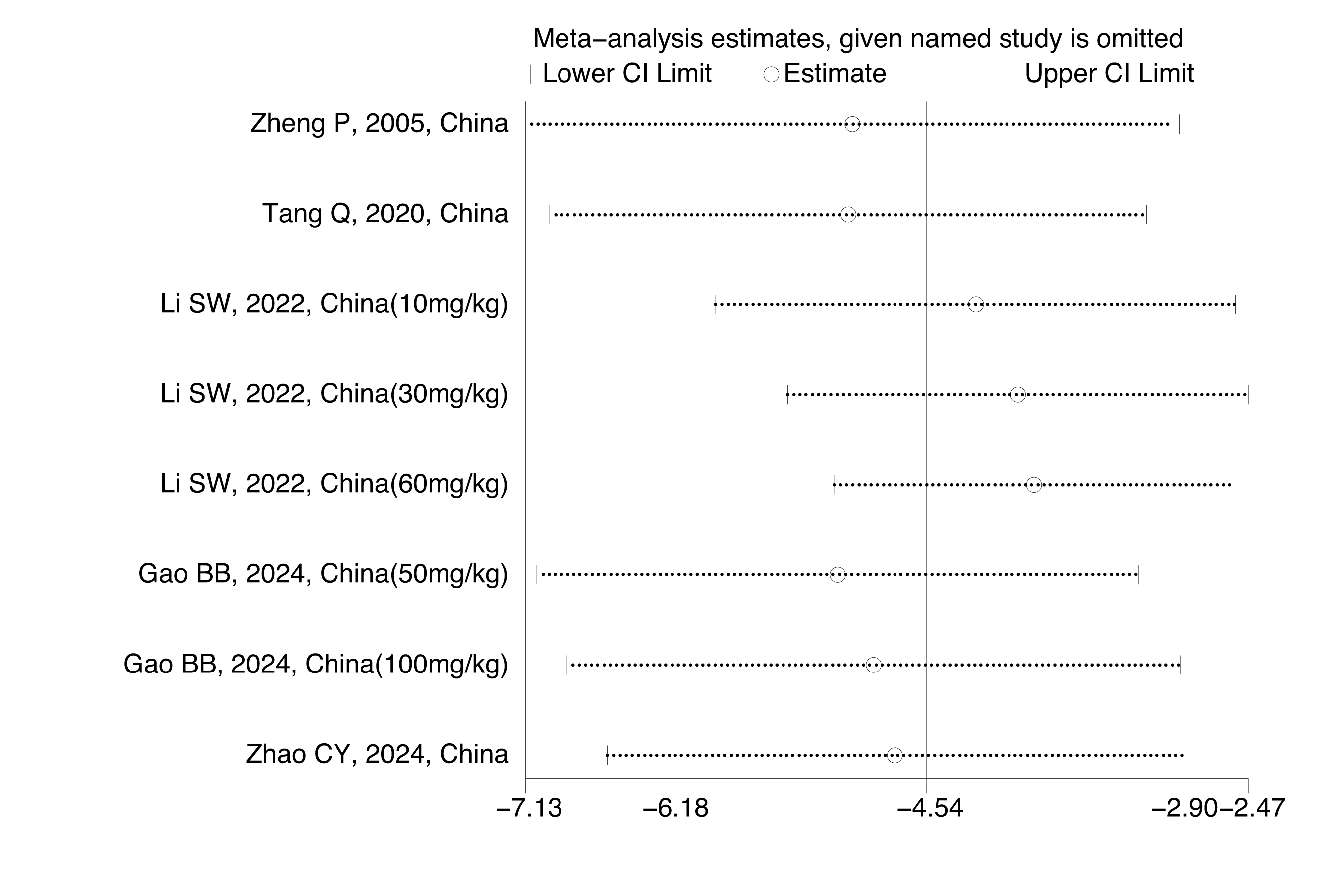


D


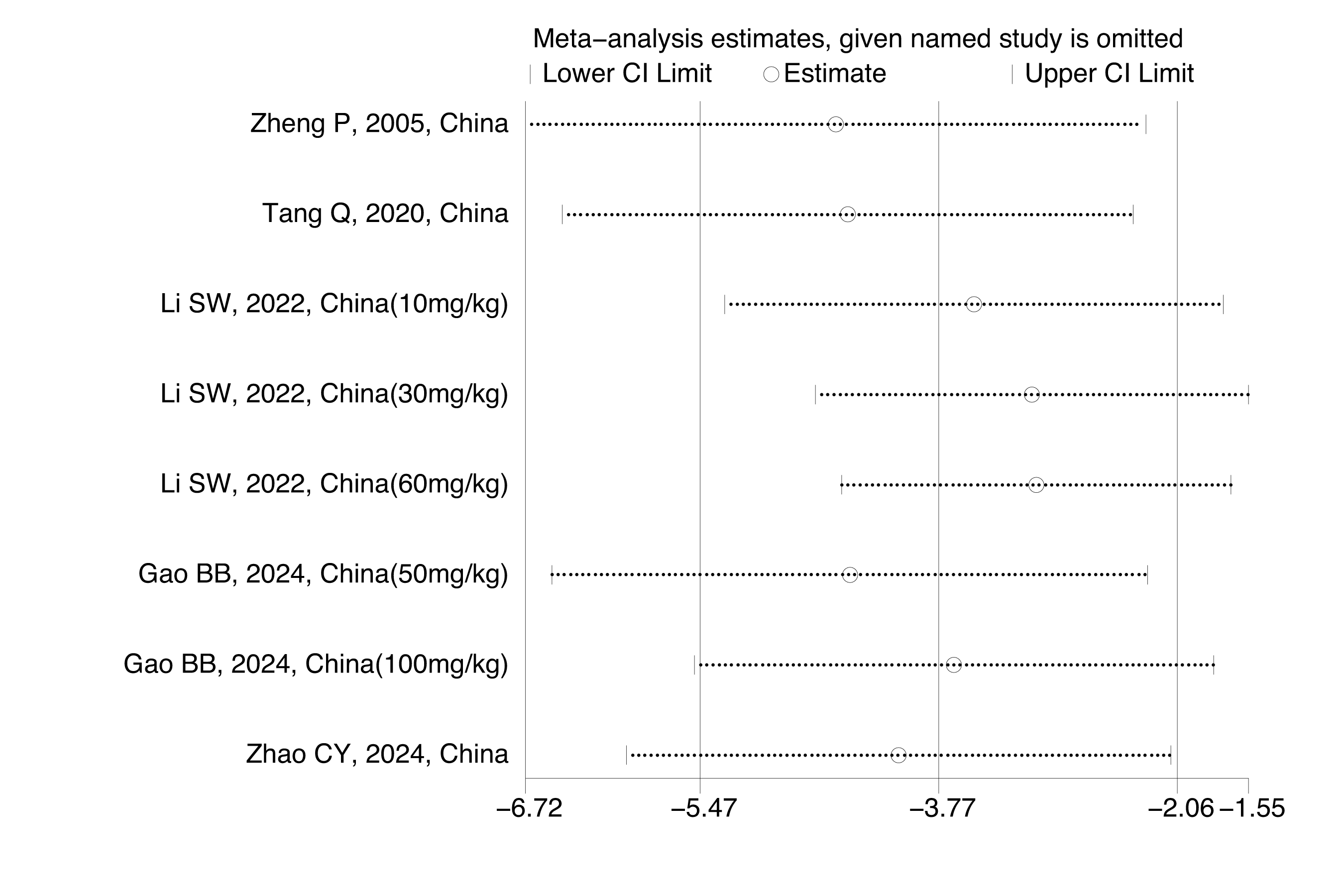


E


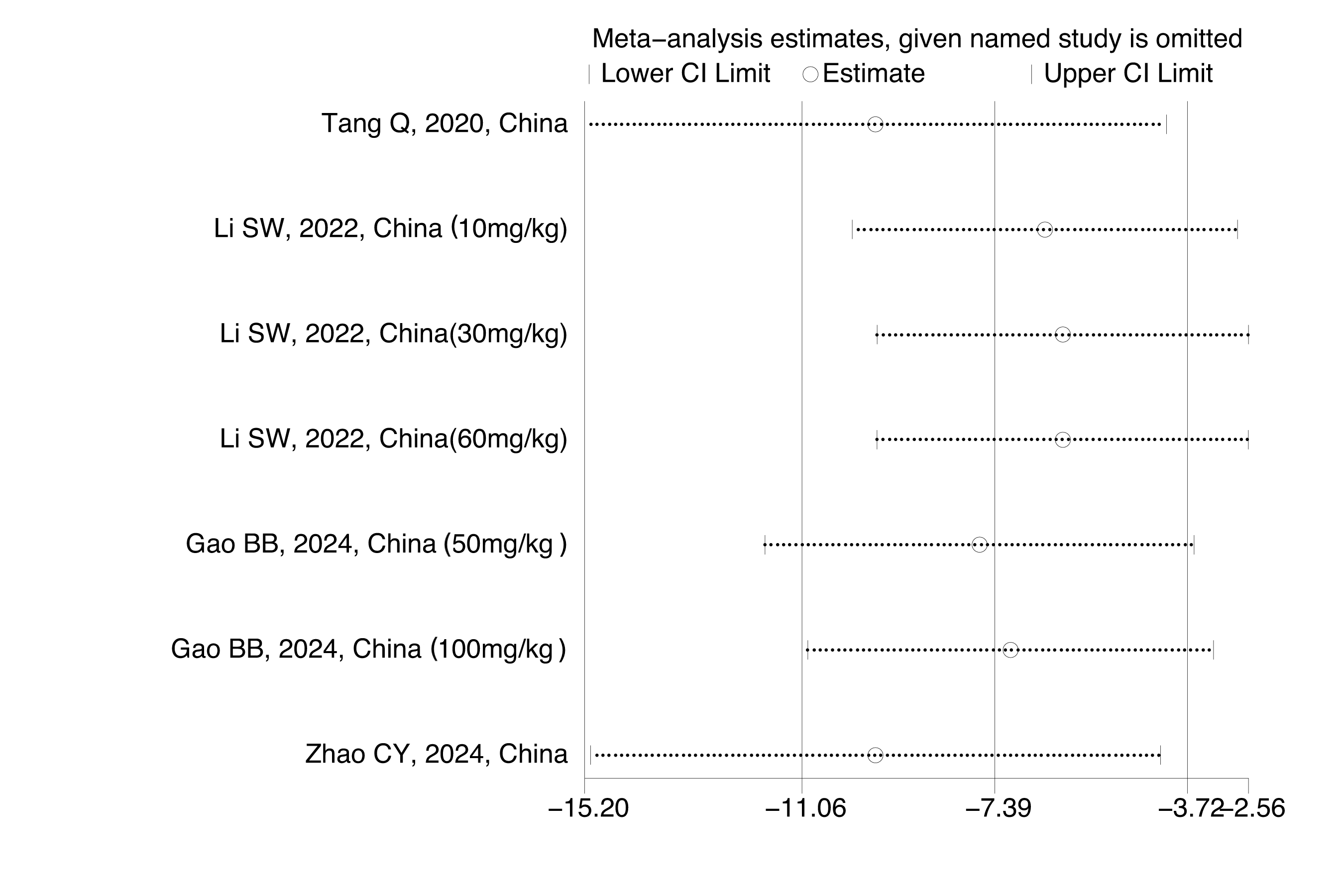


F


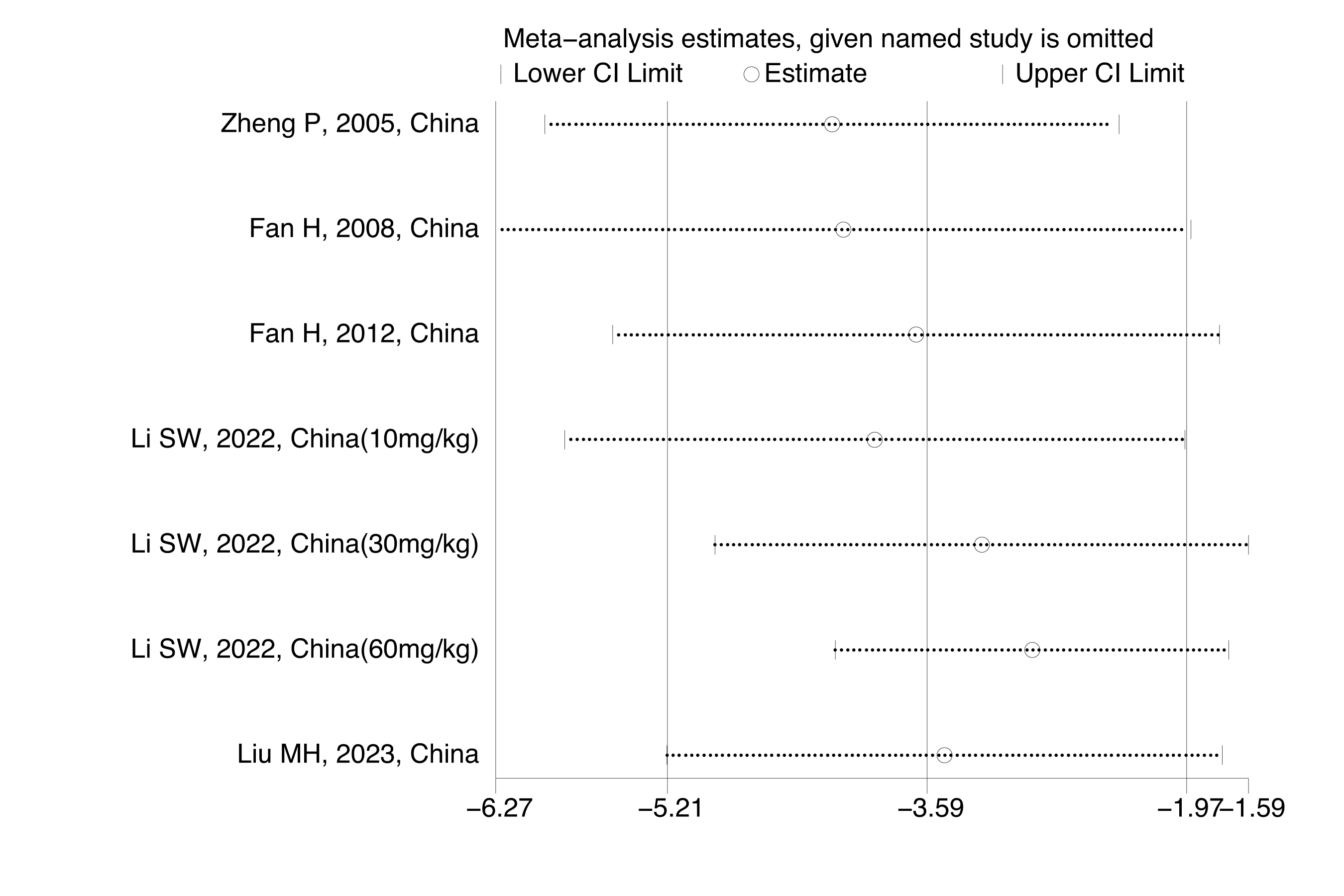


G


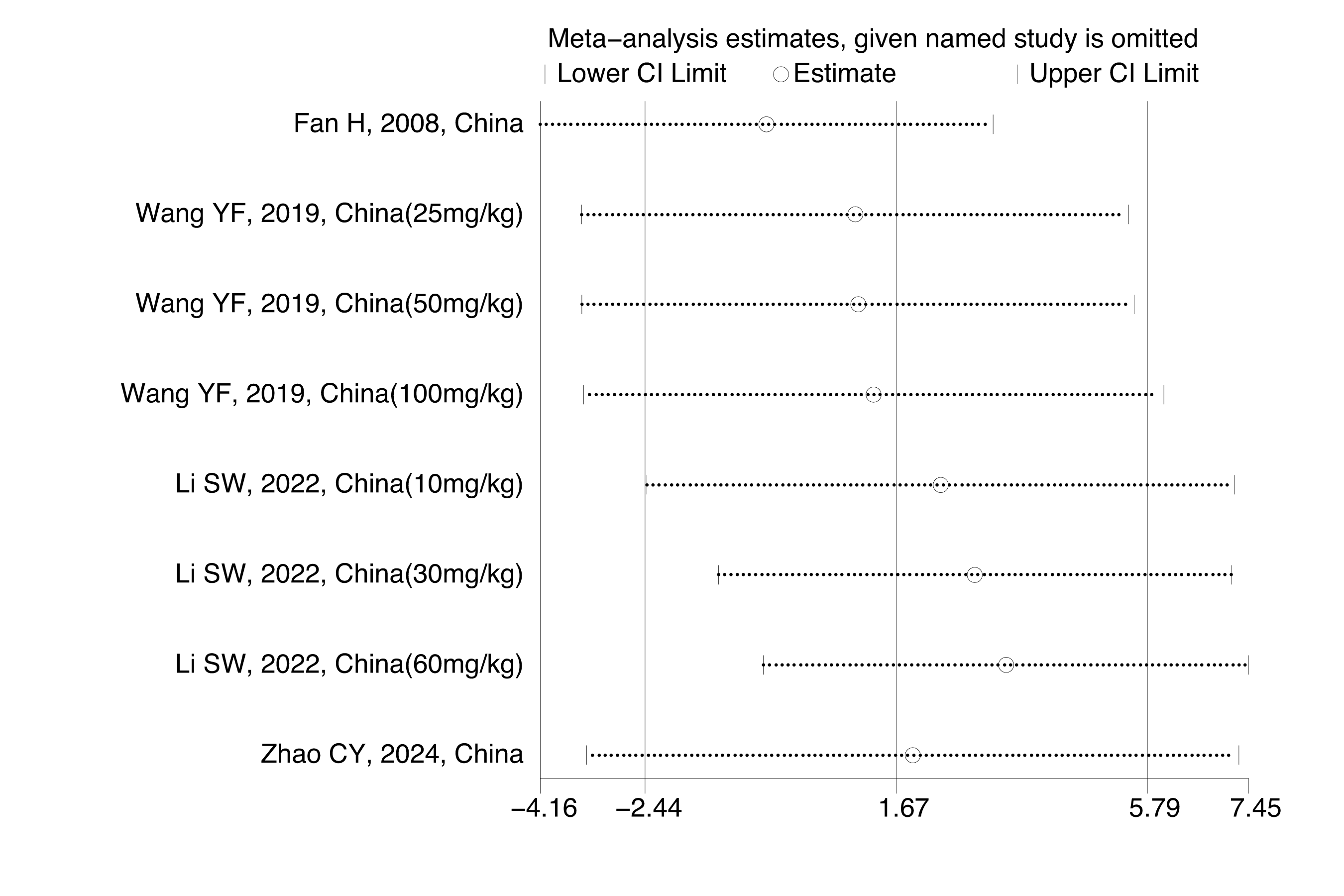


H


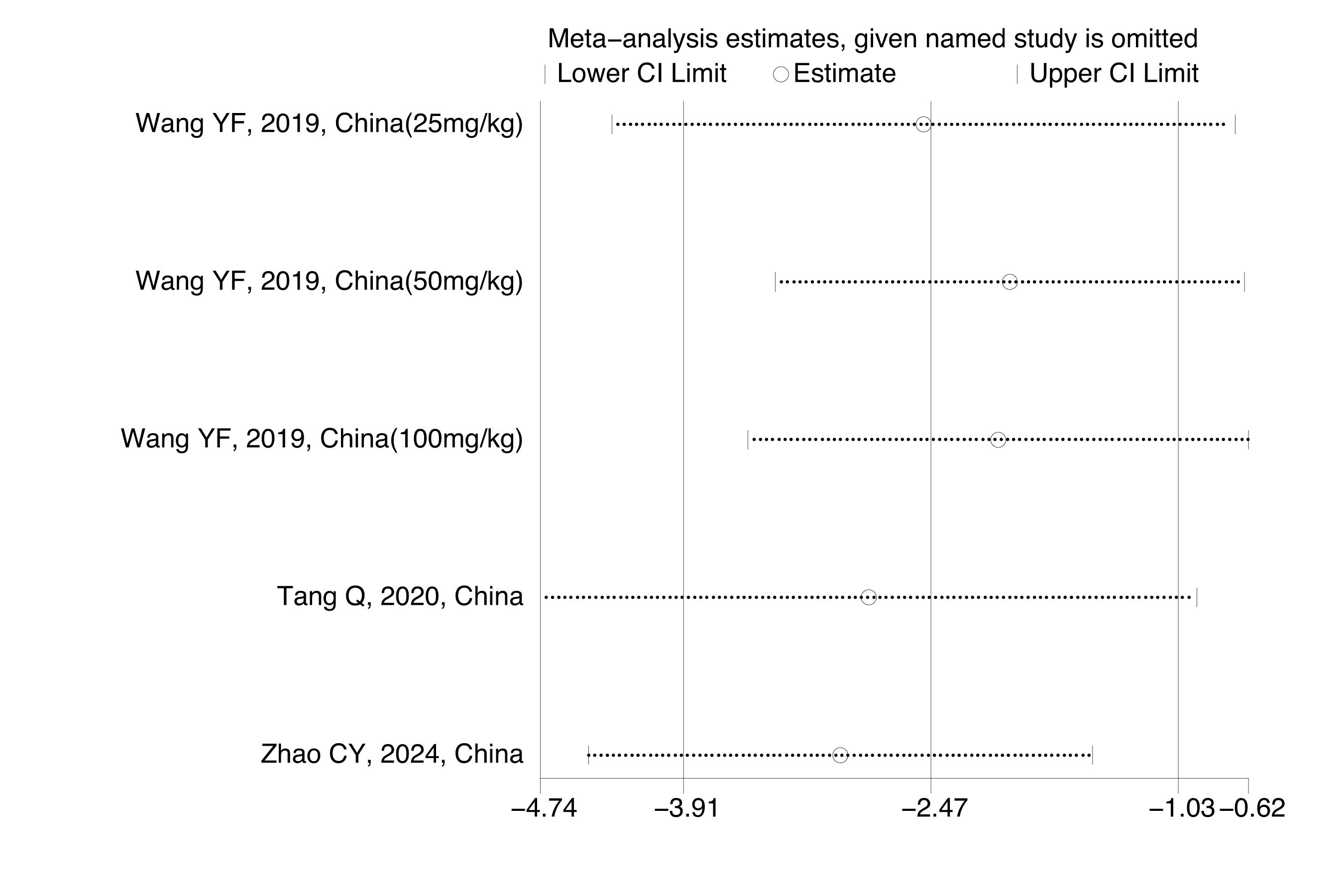


I


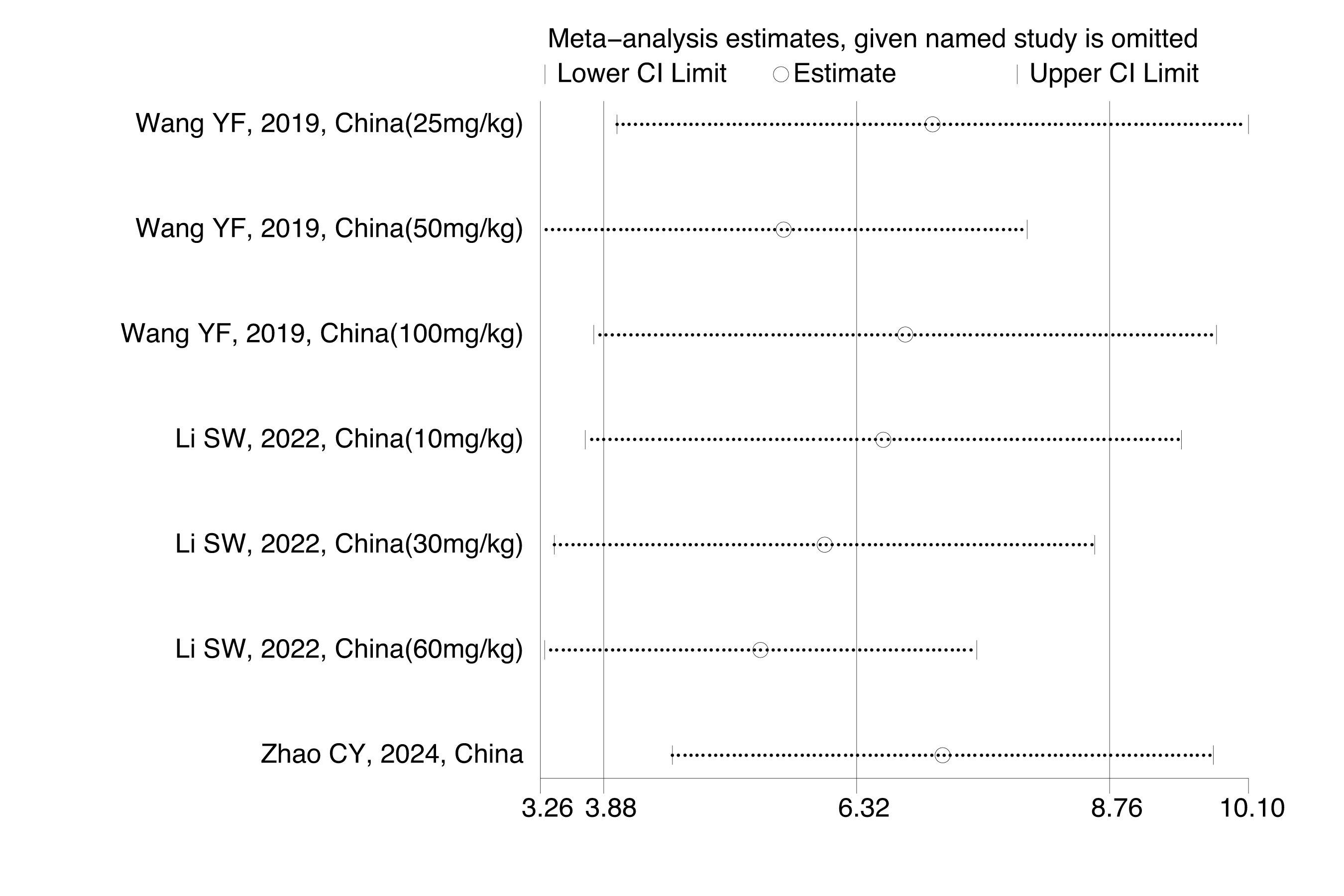


J


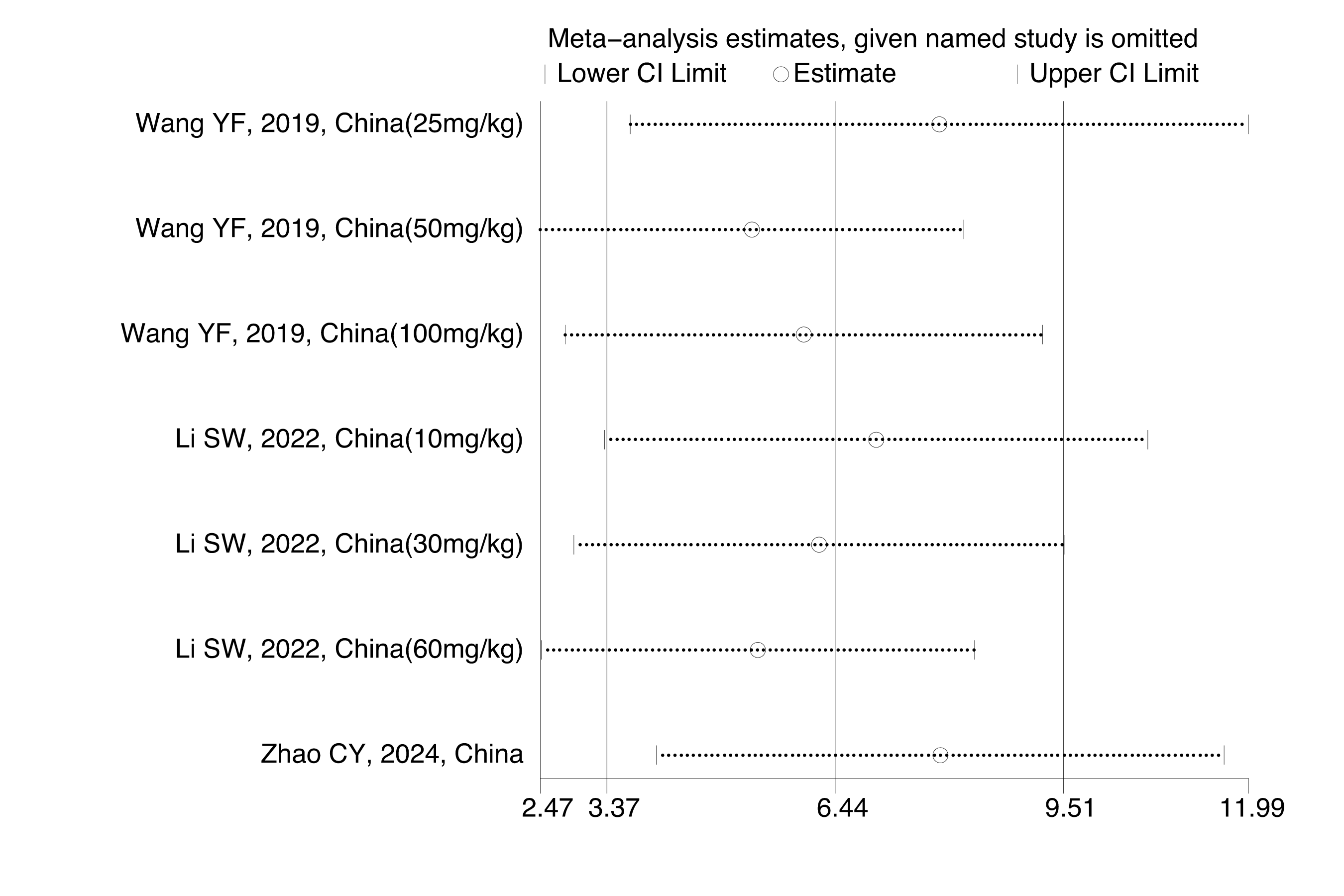


**Supplementary Figure 3.** Sensitivity analysis of histopathological score (HS), colon length, tumor necrosis factor-α (TNF-α), interleukin-6 (IL-6), interleukin-1β (IL-1β), nuclear factor-κB (NF-κB), interleukin-10 (IL-10), myeloperoxidase (MPO), zonula occludens-1 (ZO-1) and occludin. **(A)** Sensitivity analysis of HS; **(B)** Sensitivity analysis of colon length; **(C)** Sensitivity analysis of TNF-α; **(D)** Sensitivity analysis of IL-6; **(E)** Sensitivity analysis of IL-1β; **(F)** Sensitivity analysis of NF-κB; **(G)** Sensitivity analysis of IL-10; **(H)** Sensitivity analysis of MPO; **(I)** Sensitivity analysis of ZO-1; **(J)** Sensitivity analysis of occluding.
