# Supplementary material for: Oxymatrine for inflammatory bowel disease in preclinical studies: a systematic review and meta-analysis
Source: Front Med (Lausanne). 2025 Apr 30;12:1542953. doi: 10.3389/fmed.2025.1542953 (PMC12075229; doi:10.3389/fmed.2025.1542953)
Supplement: Supplementary file 4 [file Supplementary_file_3.docx]

**
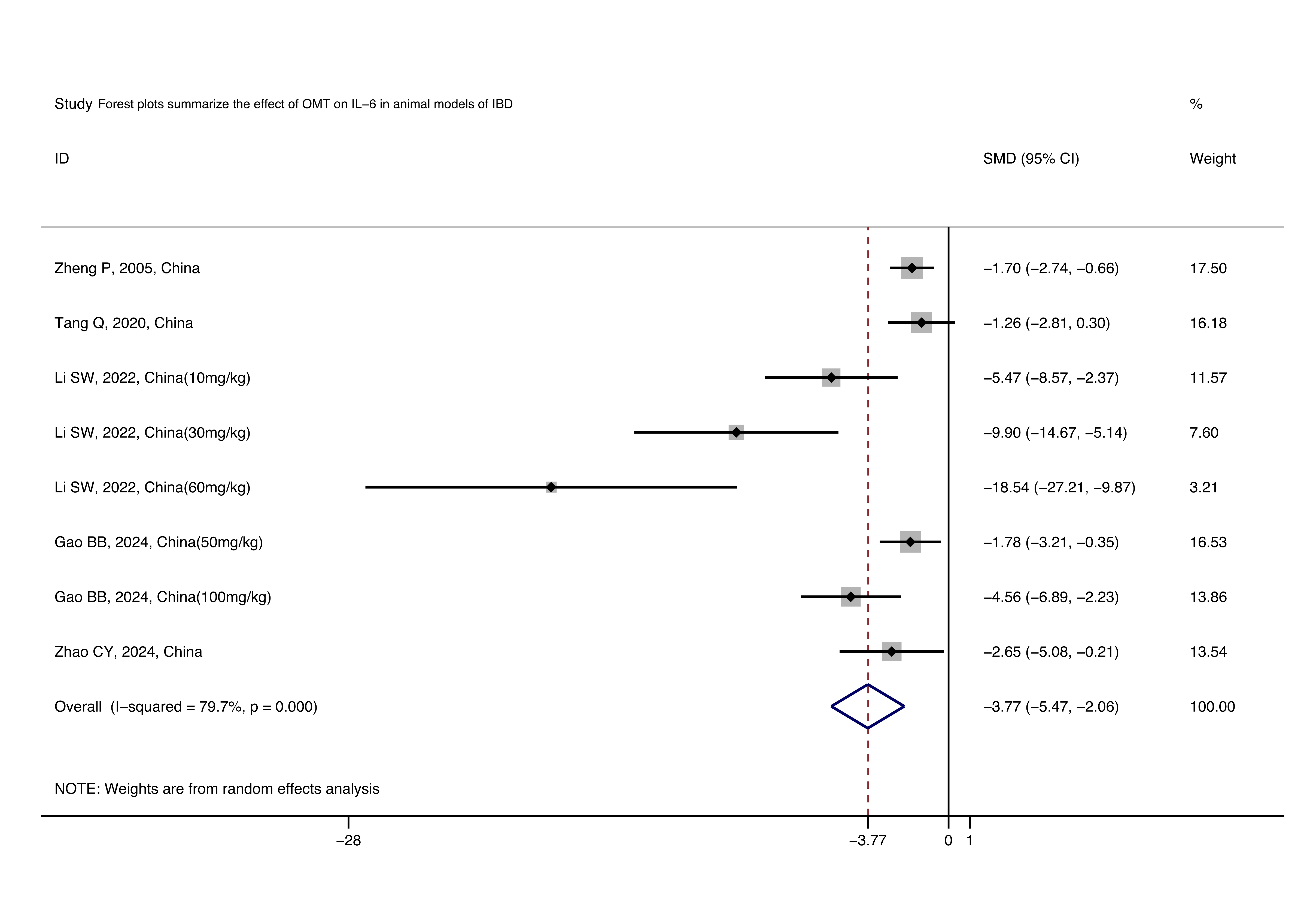
**

**Supplementary Figure 4.** Effect of oxymatrine on IL-6.

**
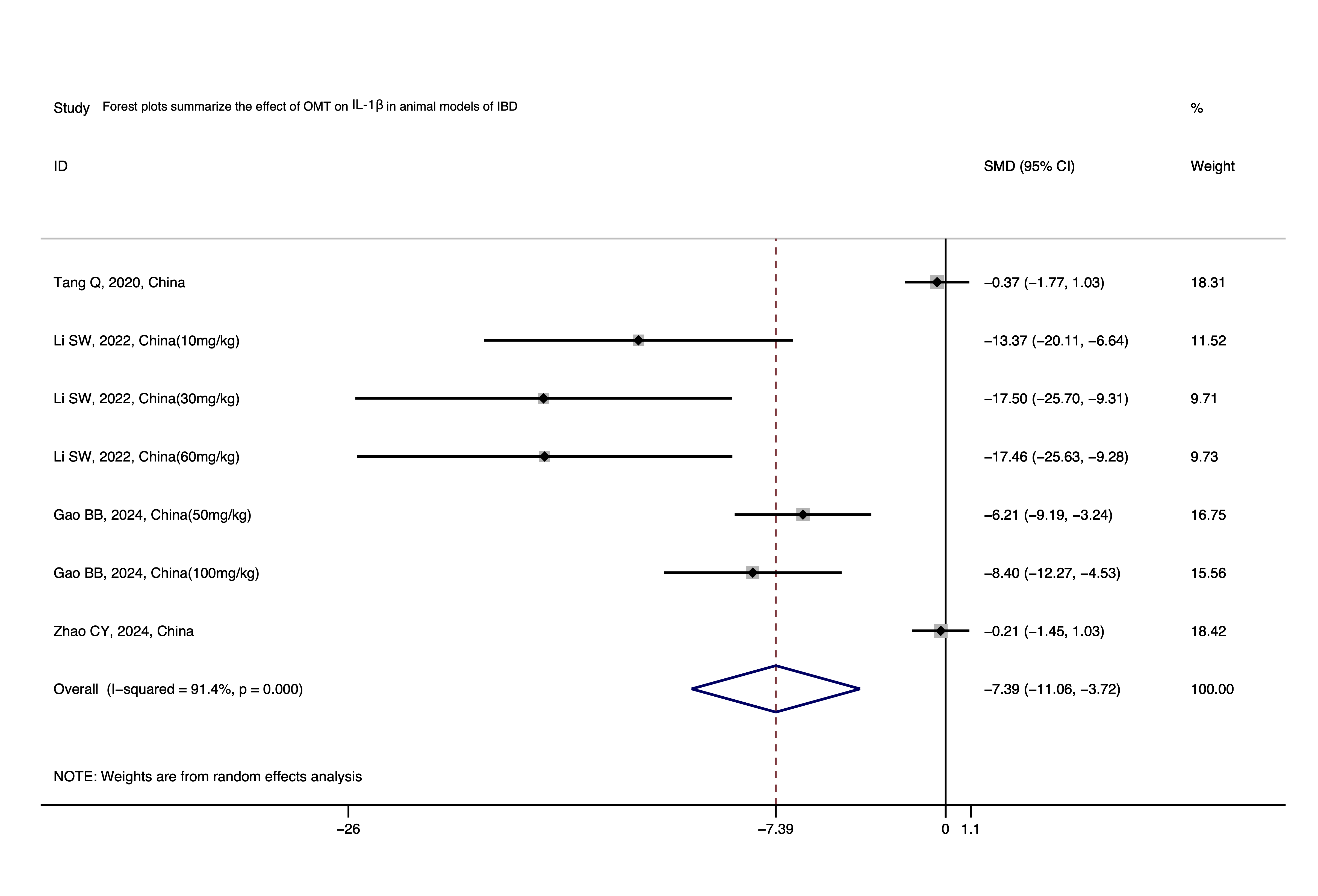
**

**Supplementary Figure 5.** Effect of oxymatrine on IL-1β.


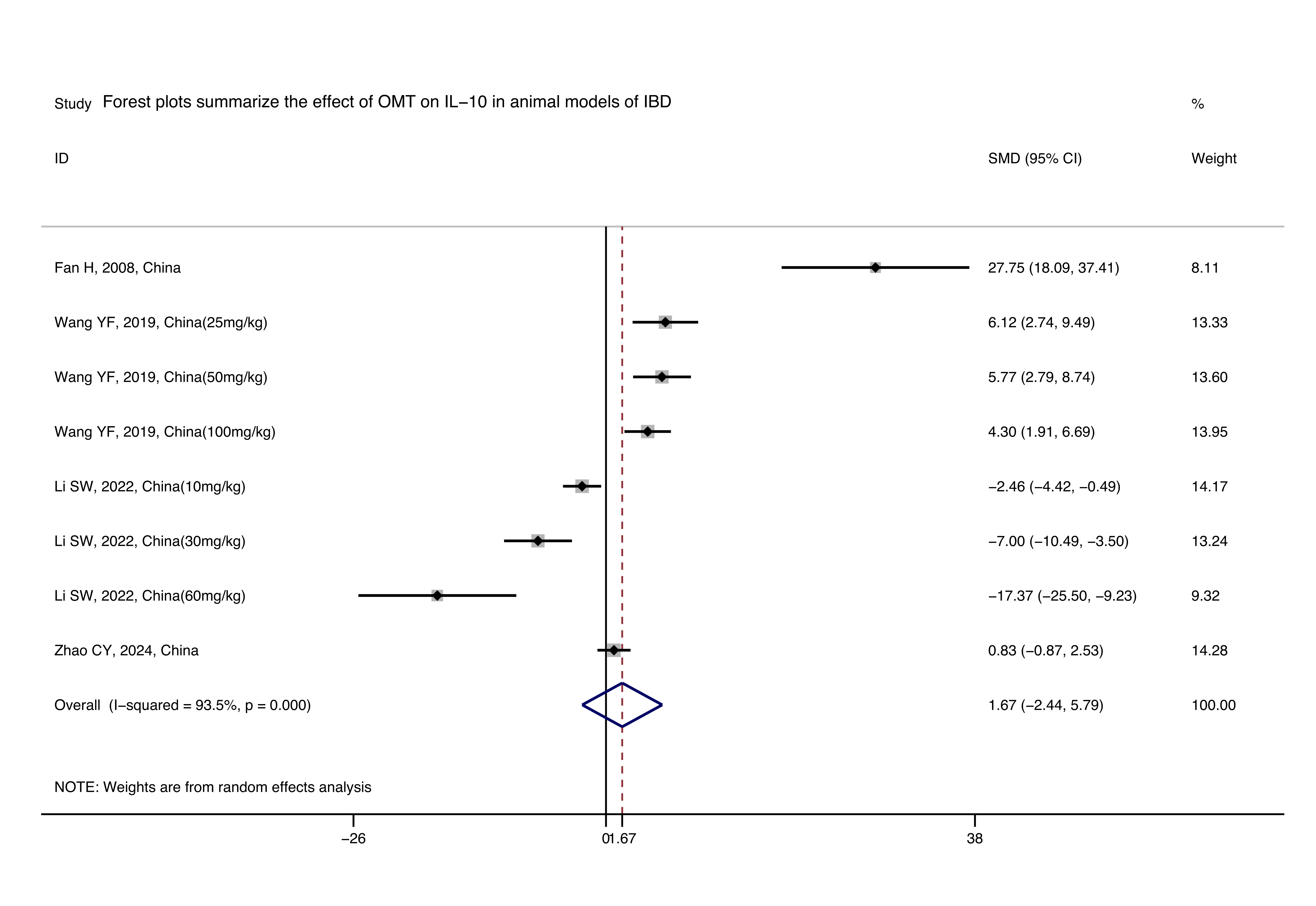


**Supplementary Figure 6.** Effect of oxymatrine on IL-10.


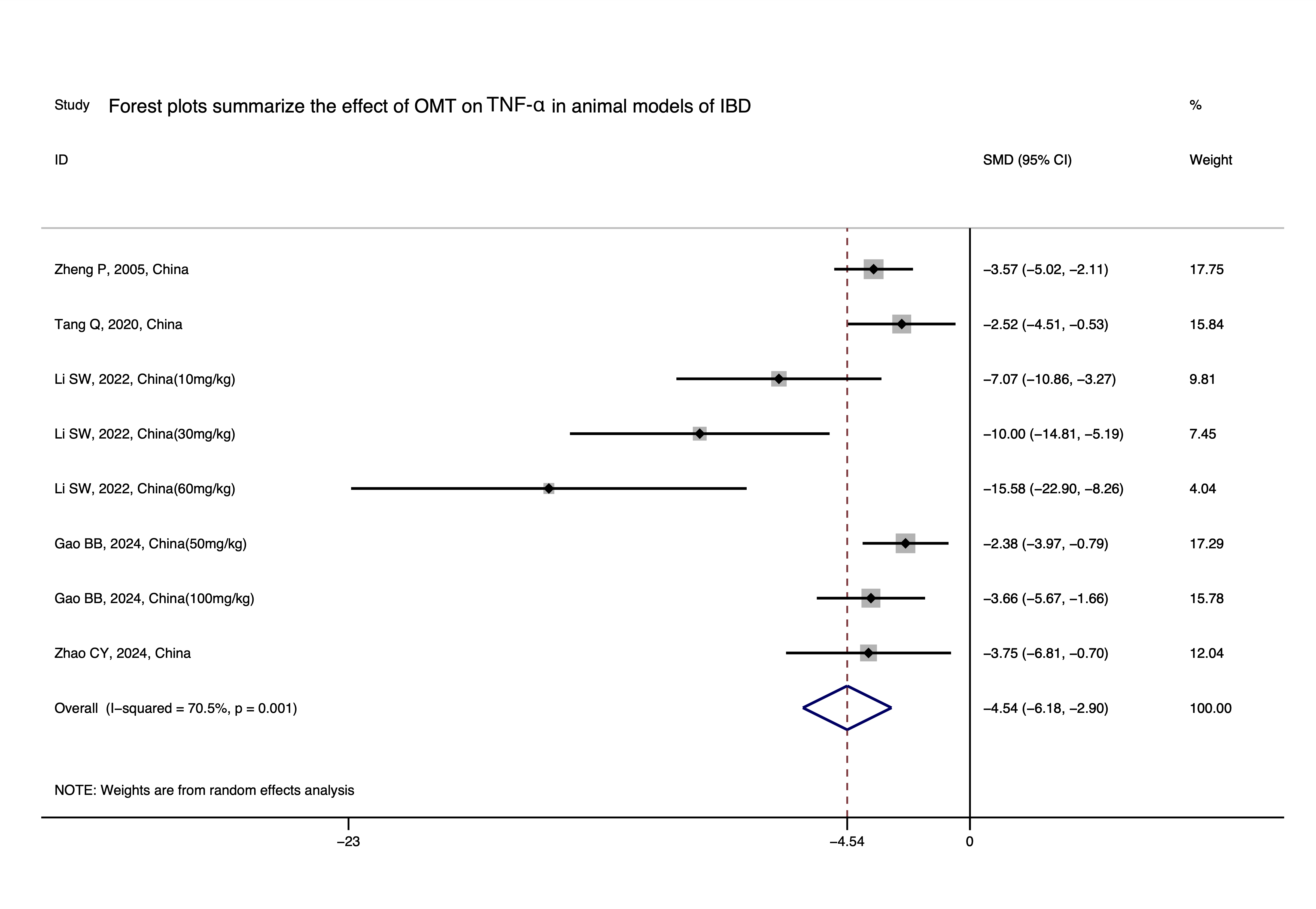


**Supplementary Figure 7.** Effect of oxymatrine on TNF-α.


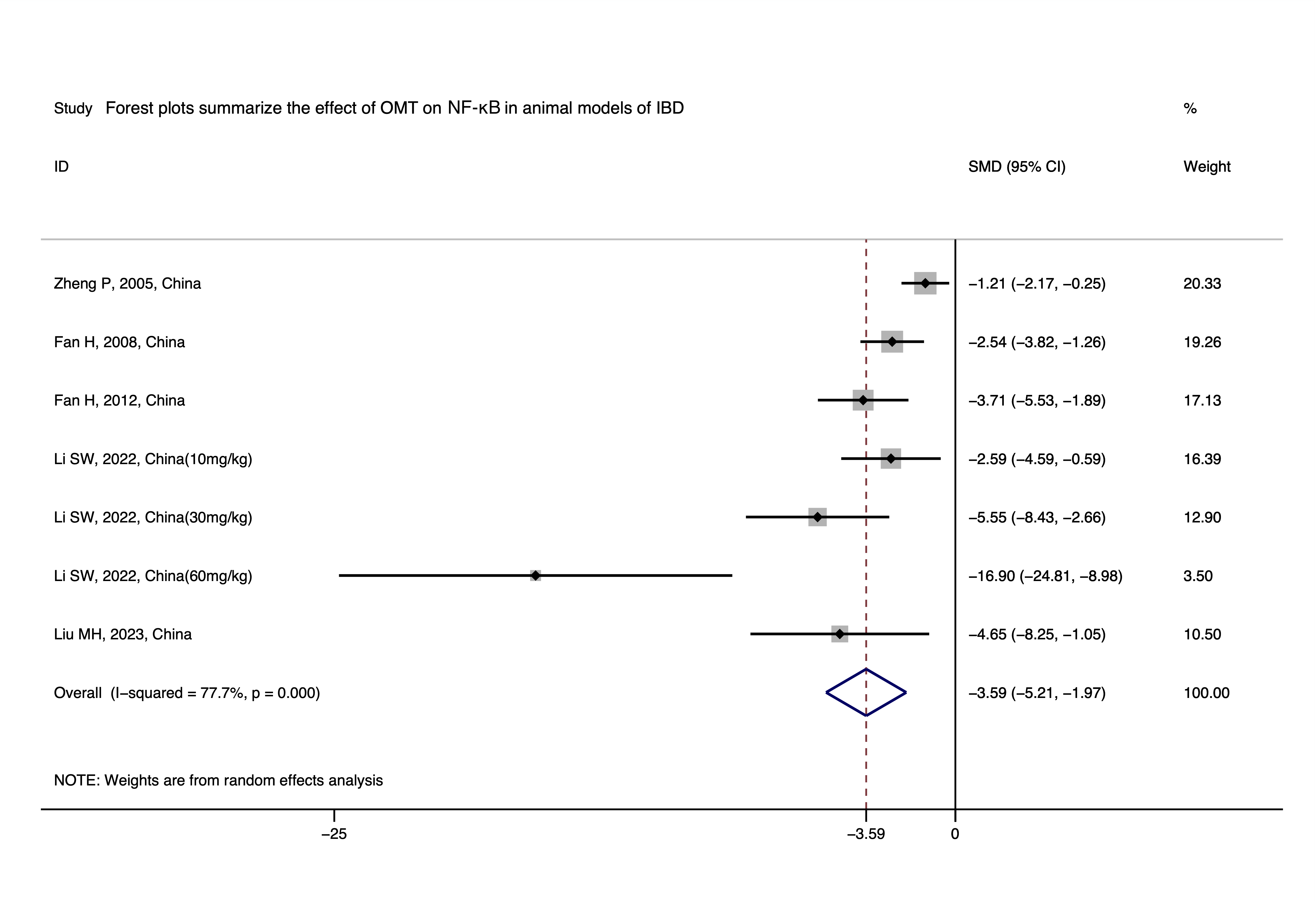


**Supplementary Figure 8.** Effect of oxymatrine on NF-κB.


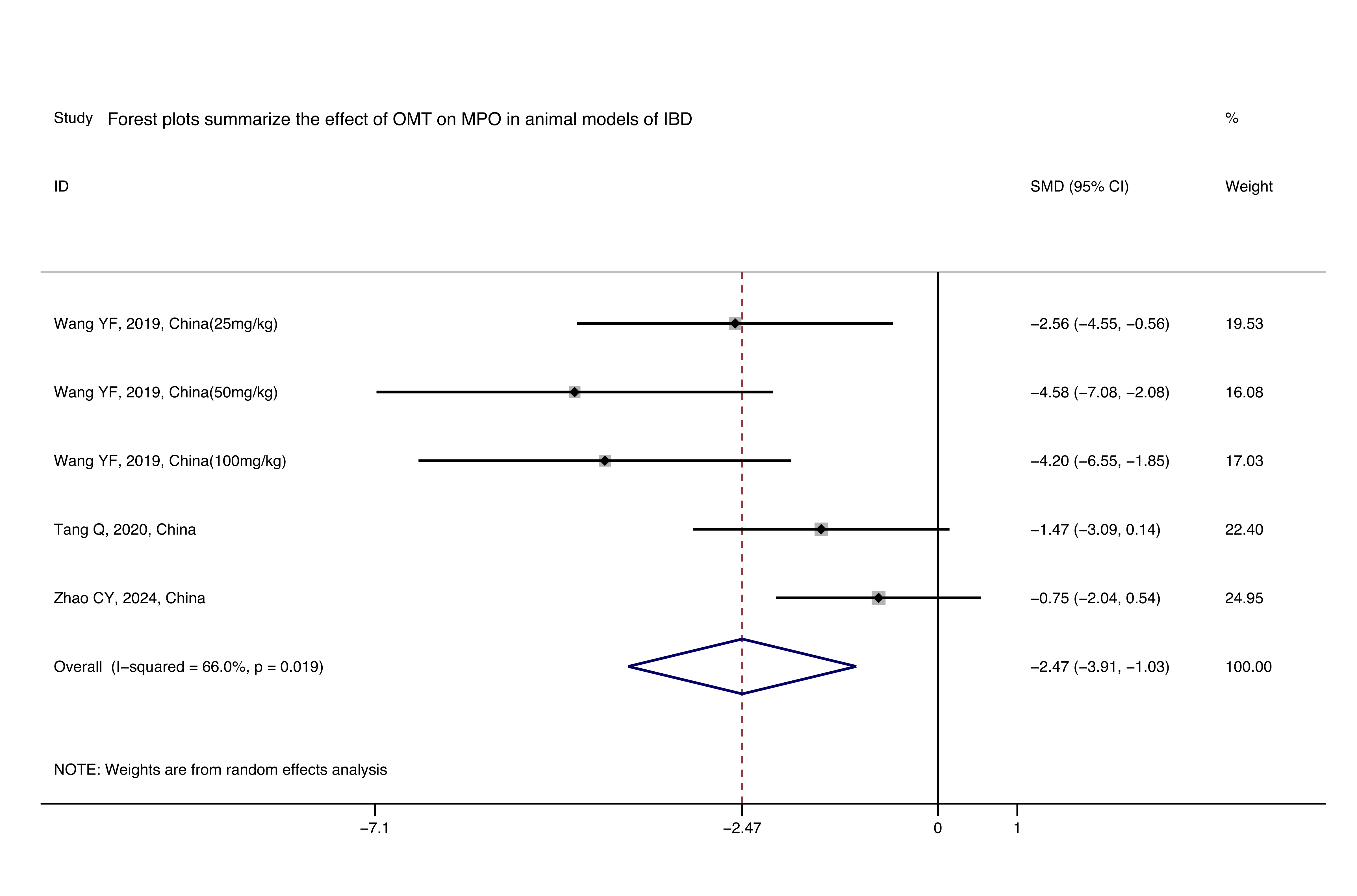


**Supplementary Figure 9.** Effect of oxymatrine on MPO.


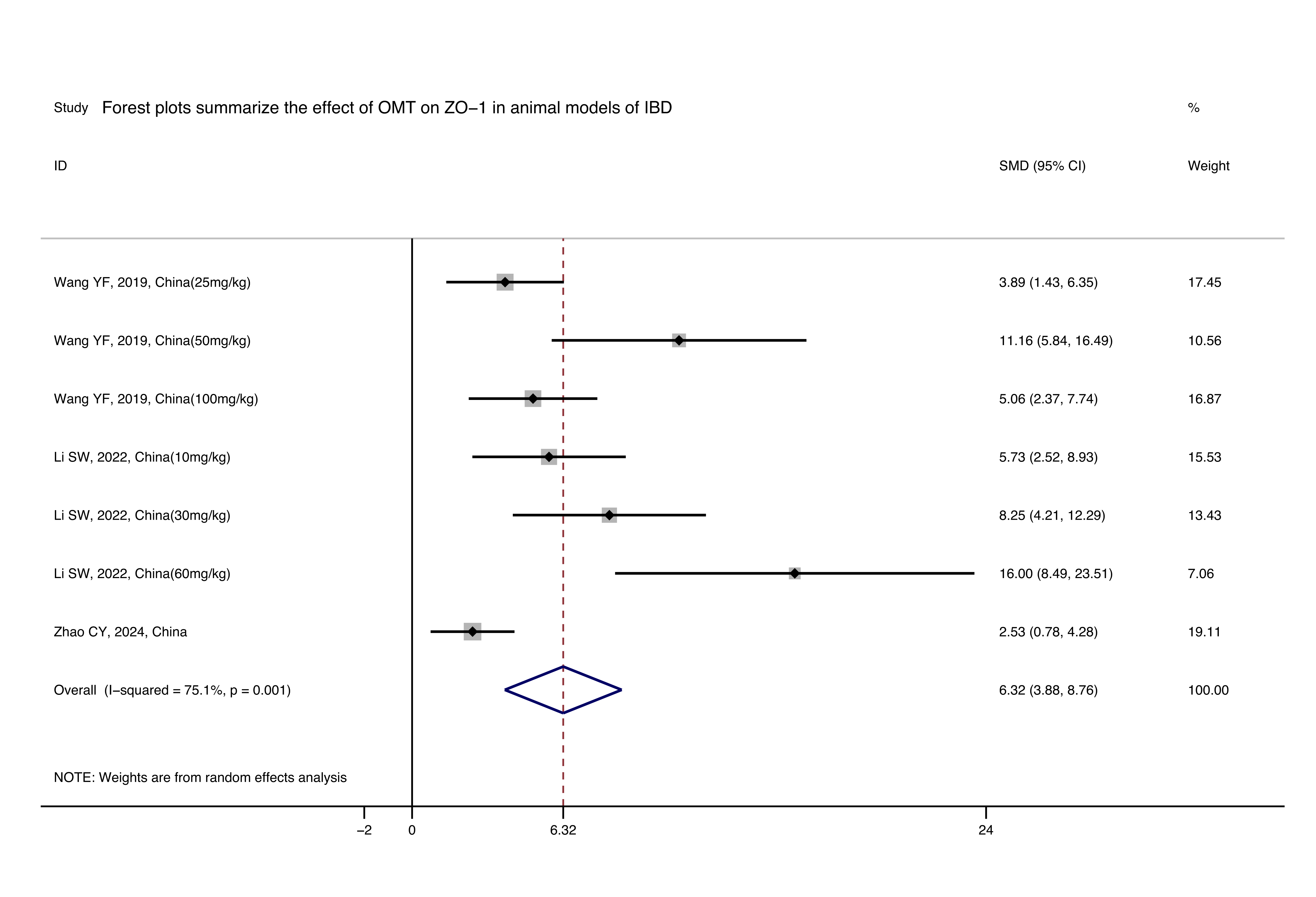


**Supplementary Figure 10.** Effect of oxymatrine on ZO-1.


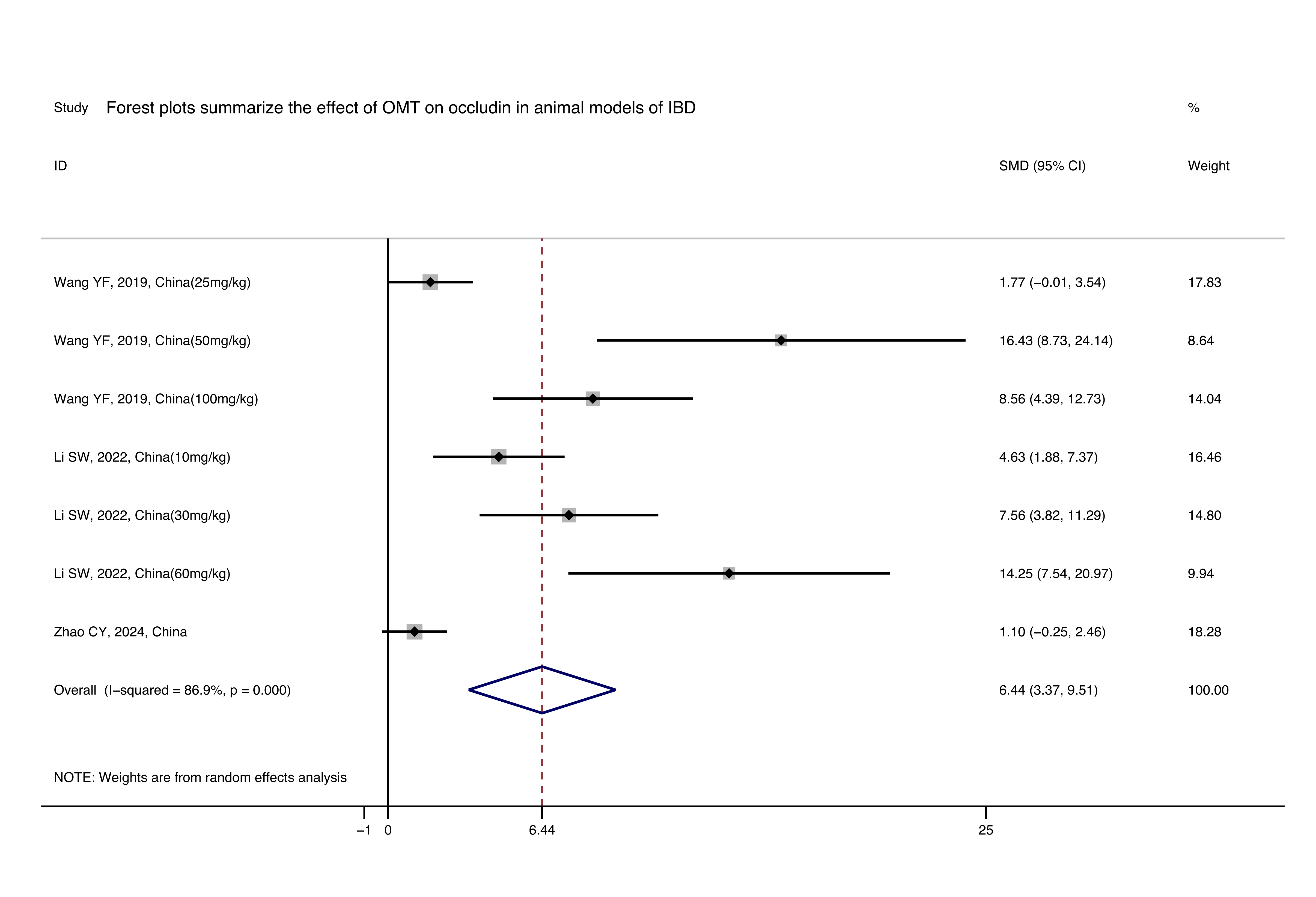


**Supplementary Figure 11.** Effect of oxymatrine on occludin.
